# A controversial issue: Can mitochondria modulate cytosolic calcium and contraction of skeletal muscle fibers?

**DOI:** 10.1085/jgp.202213167

**Published:** 2022-07-18

**Authors:** Carlo Reggiani, Lorenzo Marcucci

**Affiliations:** 1 Department of Biomedical Sciences, University of Padova, Padova, Italy; 2 Science and Research Center Koper, Institute for Kinesiology Research, Koper, Slovenia; 3 Center for Biosystems Dynamics Research, RIKEN, Suita, Japan

## Abstract

Mitochondria are characterized by a high capacity to accumulate calcium thanks to the electrochemical gradient created by the extrusion of protons in the respiratory chain. Thereby calcium can enter crossing the inner mitochondrial membrane via MCU complex, a high-capacity, low-affinity transport mechanism. Calcium uptake serves numerous purposes, among them the regulation of three dehydrogenases of the citric cycle, apoptosis via permeability transition, and, in some cell types, modulation of cytosolic calcium transients. This Review is focused on mitochondrial calcium uptake in skeletal muscle fibers and aims to reanalyze its functional impact. In particular, we ask whether mitochondrial calcium uptake is relevant for the control of cytosolic calcium transients and therefore of contractile performance. Recent data suggest that this may be the case, at least in particular conditions, as modified expression of MCU complex subunits or of proteins involved in mitochondrial dynamics and ablation of the main cytosolic calcium buffer, parvalbumin.

## Teaser

In skeletal muscle fibers, mitochondria calcium uptake is relevant for metabolic activation, trophic regulation, and apoptosis. It is still debated whether mitochondria can modulate cytosolic calcium transients and contractile performance. Here, we discuss evidence in favor or against this possibility.

## Introduction

A recent paper by Brad Launikonis and coworkers (Lamboley et al., 2021) has provided the first direct estimation of the amount of calcium that can be stored in the mitochondria of skeletal muscle fibers. At rest, in a mouse fast fiber, the total mitochondrial calcium concentration corresponding to 1.2 mmoles/L_mito_ was found, almost 5,000 times greater than the concentration of free Ca^2+^ in the matrix, 250 nmoles/L_mito_. These results point to a high buffering power of mitochondrial matrix, in contrast with previous observations of small amount of calcium stored in mitochondria of skeletal muscle (Fryer and Stephenson, 1996; Lamboley et al., 2015), and raise the mitochondrial storing capacity (60 μmoles/L_fiber_) to a level comparable to the amount of calcium mobilized during contractile activity (>300 μmoles/L_fiber_; Baylor and Hollingworth, 2007; Baylor and Hollingworth, 2012; Holash and MacIntosh, 2019). The new determination of the storage capacity of the mitochondria reopens the debate as to their possible contribution to the modulation of the cytosolic calcium transients and contractile responses in skeletal muscle fibers. However, the requirements to fulfill such task are not only the ability to store calcium but also the kinetics of taking up calcium, binding it to the buffers in the matrix and release it with a timing compatible with the rapid release and reuptake from and to SR. The following sections are aimed to discuss quantitatively these points.

Here and hereinafter, the concentrations of free ionized calcium (Ca^2+^) or of total calcium are expressed with reference to 1 liter of the total fiber volume (L_fiber_) or of mitochondrial volume (L_mito_). A ratio 1:20 links the two volume references, under the assumption that, in murine fast fibers, mitochondria volume is 5% of fiber volume (see, e.g., Butera et al., 2021). In addition, the relation between mitochondrial volume and mitochondrial proteins is based on the proportion of 2.6 μl per 1 mg protein (Schwerzmann et al., 1989).

## Mitochondrial calcium uptake: Historic milestones

The first demonstration that mitochondria accumulate calcium was given 60 yr ago by Deluca and Engstrom (1961) and by Vasington and Murphy (1962). Kidney isolated mitochondria could store in 10–20 min large amount of calcium, in the order of 1–3 μmoles/mg of mitochondrial proteins. This finding appeared immediately consistent with the chemiosmotic theory, in particular with the concept of an energetically favorable electrochemical gradient that drives the rapid influx of cations across the inner mitochondrial membrane (IMM). In the same years, mitochondria were found to be able to accumulate large amounts of inorganic phosphate (Brierley et al., 1962, Brierley et al., 1963, cardiac muscle).

The determination of the kinetics parameters on isolated mitochondria preparations revealed that Ca^2+^ uptake mechanism is characterized by high capacity, cooperativity, and low affinity (see a compilation of published values in Table 1.) The large diversity among the parameter values might depend partly on the diversity among tissues and partly on the experimental conditions. Among the cytosolic factors that influence mitochondrial Ca^2+^ uptake, cytosolic free Mg^2+^ concentration has a special relevance since it can strongly depress the mitochondrial Ca^2+^ uptake (see Hutson et al., 1976, liver mitochondria; Favaron and Bernardi, 1985, heart, kidney, and liver) at its physiological level close to 1 mM (Csernoch et al., 1998). Inorganic phosphate, in contrast, can speed up Ca^2+^ transport rate (Wei et al., 2012). Importantly, a threshold for the calcium transport was discovered, as data indicated that virtually no uptake occurs with Ca^2+^ concentrations in the reaction medium below 200–300 nM (Brinley et al., 1978, squid axon; see also Magnus and Keizer, 1997, pancreas β cells).

**Table 1.** Kinetic parameters determined in isolated mitochondrial preparations from various cell types.

reference	Tissue	T°C	V_max_ nmol/mg/min	V_max_ mmol/L_mito_/s	Affinity K_0.5_ μM	nH	Threshold nM
Bragadin et al., 1979	Liver	20	900	5.76		2	
Hutson et al., 1976	Liver	25	640	4.10	3.1		
Vinogradov and Scarpa, 1973	Liver	24	480–780	3.07–4.99	55–70	1.63	
Reed and Bygrave, 1975	Liver	0	19	0.12	2-4	1.7	
McMillin-Wood et al., 1980	Heart	30	577–1750	3.69–11.21	74–189	1	
Scarpa and Graziotti, 1973	Heart	26	480–840	3.07–5.38	30–90	2	
Sembrovich et al., 1985	Skelet. muscle	37	Fast: 55	0.35	0.89	2	
		37	Slow: 158	1.01	2.51	3.5	
Brinley et al., 1978*	Squid axon	17	200	1.28			200–300

The experimental data on mitochondrial calcium uptake in nmol/mg protein/minute were converted to μmol/L_mito_/s using a factor $\Phi_{mito}=\frac{1}{60}\frac{\min}{s}\frac{10^{3}}{2.6}\frac{mg}{L_{mito}}=6.41,$ or to μmol/L_fiber_/s using a factor $\Phi_{fiber}=\frac{1}{60}\frac{\min}{s}\frac{10^{3}}{2.6}\frac{mg}{L_{mito}}0.05\frac{L_{mito}}{L_{fiber}}=0.32,$ since a ratio 1:20 links the two volume references, under the assumption that, in murine fast fibers, mitochondria volume is 5% of fiber volume (see Butera et al., 2021) and 1 mg protein corresponds to 2.6 μl of mitochondria (Schwerzmann et al., 1989). *, intact axon preparation.

Sodium in the perfusing medium was found to activate the efflux of calcium from isolated mitochondria leading to the identification of the sodium-calcium counter-transport as a major way to export calcium from mitochondria (Carafoli et al., 1974; Crompton et al., 1976). A direct proton–calcium exchange is present only in some cell types and sodium–calcium exchange is activated by sodium gradient created by the sodium–proton exchange through the IMM.

The combination of a calcium uptake mechanism based on the electrochemical gradient through the IMM and of a calcium extrusion mechanism based on an electrogenic sodium–calcium exchange suggested the existence of a concentration of cytosolic calcium at which uptake and release are in balance. The balance between in and out fluxes became the basis of the model of the set-point, which supports a potential role of mitochondria in regulating cytosolic Ca^2+^ concentration (Nicholls, 1978, liver mitochondria). At cytosolic calcium concentrations higher than the set-point, mitochondria work as net Ca^2+^ accumulators, while net Ca^2+^ extrusion from mitochondria occurs when the cytosolic Ca^2+^ concentration is below the set-point. However, the set-point concentrations predicted by that balance were relatively high, between 0.5 and 2 μM, while the first direct determinations of resting cytosolic calcium concentrations yielded values of about 0.1 μM in most cell types (Tsien and Rink, 1980; Marban et al., 1980). Actually, the low level of cytosolic Ca^2+^ concentration in quiescent cells is expected to be determined by calcium exchanges through the plasma membrane (see Friel and Tsien, 1992; Ríos, 2010).

The discovery of the calcium-dependent dehydrogenases revealed a specific function for mitochondrial calcium uptake. Denton and coworkers (Denton et al., 1972; Denton et al., 1980 demonstrated that, in cardiac mitochondria, three dehydrogenases were sensitive to calcium: pyruvate dehydrogenase, isocitrate dehydrogenase, and 2-oxoglutarate dehydrogenase, reaching full activity at 1 μM free Ca^2+^ concentration. Actually, the activation of pyruvate dehydrogenase depends on the dephosphorylation of the catalytic subunit by a Ca^2+^-dependent phosphatase, whereas α-ketoglutarate and isocitrate dehydrogenases are directly activated by Ca^2+^ binding. On this ground, the main physiological role of mitochondrial calcium uptake was identified in the regulation of ATP production in relation to cytosolic calcium transients. The mechanism was considered particularly relevant for cardiac and skeletal muscles where the ATP production needs to be rapidly adjusted to functional requirements.

The metabolic activation induced by calcium entry in mitochondria causes a not negligible side effect in the increased generation of reactive oxygen species (ROS). In physiological conditions, mitochondrial Ca^2+^ uptake contributes to ROS generation, by impinging on Krebs cycle enzymes and therefore activating electron transport chain activity, where superoxide is mainly produced by complex I and complex III (Turrens, 2003; Feno et al., 2019). Superoxide (O_2_^−^) is produced by partial reduction of molecular oxygen, then hydrogen peroxide (H_2_O_2_) is formed by superoxide dismutase (SOD), and finally transformed in water by glutathione peroxidase, peroxiredoxin, and catalases. The balance between ROS production and scavenging modulates their biological effects. Additionally, superoxide can react with NO and generate peroxynitrite. Importantly, ROS can modulate activity of molecules involved in calcium homeostasis. RyR nitrosylation enhances leakage (Aracena-Parks et al., 2006; Sun et al., 2013), thus raising cytosolic Ca^2+^ levels, while mitochondrial calcium uniporter (MCU) oxidative modifications increase its permeability to calcium (Dong et al., 2017). In both cases, positive feed-back responses are induced: more calcium enters mitochondria and more ROS are generated (Canato et al., 2019). An intriguing example of reciprocal ER–mitochondria communication is the release of Ca^2+^ by IP_3_R in response to ROS-based signals released by mitochondria during flicker activity (Booth et al., 2021).

The discovery (Hunter et al., 1976; Crompton et al., 1987) that in cardiac mitochondria a permeability transition of the IMM is induced by high concentration of calcium in the matrix revealed that another way of calcium efflux was possible, a way which is mostly but not always irreversible. Transient changes in permeability of the IMM (flickering) can lead to Ca^2+^ release from mitochondria when a suitable concentration gradient exists between matrix and cytosol and thus contributes to the inner mitochondrial Ca^2+^ homeostasis. In contrast, a prolonged change in permeability constitutes a point of no return and drives the cell towards apoptosis or necrosis, depending on the residual intracellular ATP availability. The discovery of the link between the permeability transition and cell death allowed the completion of a model with three major functions for mitochondrial calcium uptake: metabolic regulation, control of cytosolic calcium, and cell death. This view was well validated and accepted for liver, heart, and brain. As to cardiomyocytes, doubts were raised whether mitochondrial calcium could follow the fast systo-diastolic oscillations of cytosolic calcium. In contrast, the essential contribution of mitochondria and permeability transition in ischemic myocardial injury was widely accepted and this implied a role of mitochondrial calcium overload in cardiomyocyte death (Miyata et al., 1991; Griffiths and Halestrap, 1995).

In the nineties, a paramount advancement was the design of the mitochondrial matrix calcium indicators or calcium sensors, indo1 (Miyata et al., 1991), aequorin (Rizzuto et al., 1992), and rhod2 (Jou and Sheu, 1994). The experiments with calcium sensors removed all possible doubts that mitochondrial calcium was not able to closely follow the kinetics of cytosolic calcium even on a short time scale. Interestingly, the design of specifically targeted aequorin (Rizzuto et al., 1992) allowed the detection of parallel cytosolic and mitochondrial calcium variations also in cultured myotubes (Brini et al., 1997). The generation of cameleons, i.e., calcium probes based on FRET in proteins designed with GFP and calmodulin (Miyawaki et al., 1997) and targeted to mitochondrial matrix allowed direct and quantitative measurements of free mitochondrial Ca^2+^ (Arnaudeau et al., 2001). In cardiomyocytes, systo-diastolic variations were detected (Jaconi et al., 2000) and in intact skeletal muscle fibers variations of free Ca^2+^ in the mitochondrial matrix synchronous with the cytosolic transients were observed (Rudolf et al., 2004; Scorzeto et al., 2013).

The correct calibration of the probes allowed reliable measurements of the free Ca^2+^ concentration in the mitochondrial matrix. While values between 100 and 200 nM were found in various cell types at rest, significant discrepancy emerged in the concentrations induced by electrical stimulation or by administration of chemical or pharmacologic compounds. Values in the range of 500–1,500 nM were measured with Rhod 2 in astrocytes (Jou et al., 1996) and in chromaffine cells (Babcock et al., 1997), in synaptic terminals with Rhod5-N (David and Barrett, 2003), in cardiomyocytes with pericam (Robert et al., 2001), mitycam (Lu et al., 2013), cameleon (Wüst et al., 2017), and in skeletal muscle fibers with cameleon (Scorzeto et al., 2013). In contrast, much higher values, up to 10–80 μM, were measured with aequorin (Rizzuto et al., 1994; Calì et al., 2012). Several factors can contribute to such discrepancy, among them the affinity and the kinetics of the probes, the different protocols and conditions adopted for calibration and, possibly, also real difference among cells see Fernandez-Sanz et al. (2019), for a discussion. As shown by Montero et al. (2000), using aequorin as indicator, in the same chromaffin cells distinct mitochondria populations reaching peaks of matrix calcium above 100 μM or limited to 1–5 μM could be detected.

While providing an unquestionable demonstration of the synchrony between the free Ca^2+^ concentration changes in the mitochondrial matrix and in the cytosol, the direct measurements of mitochondrial calcium stressed the conflict with the low affinity of the calcium transport mechanism. The conflict was solved by the discovery of microdomains or hot spots (Rizzuto et al., 1993). Close to the calcium release sites of the endoplasmic reticulum, free Ca^2+^ reaches concentrations high enough, in the order of tens of μM, to interact with MCU and enter the mitochondria. Such optimal condition is only reached where a proximity exists between the MCU and the calcium source, virtually only for the close contact between mitochondria and intracellular calcium stores (Collins et al., 2001; Giacomello et al., 2010). The design of specific calcium probes targeted to the outer mitochondrial membrane allowed the measurement of concentrations of free Ca^2+^ up to 20–30 μM in neonatal cardiomyocytes (Drago et al., 2012) during spontaneous contractile activity and caffeine stimulation and up to 15–20 μM in Hela cells during histamine stimulation (Giacomello et al., 2010).

In the last decade, the molecular architecture of the mitochondrial calcium uptake complex has been clarified. Around the pore forming unit (MCU; De Stefani et al., 2011; Baughman et al., 2011) with its dominant negative form (MCUb; Raffaello et al., 2013) were identified the scaffold or essential mitochondrial calcium uniporter regulator (EMRE; Sancak et al., 2013), the regulatory subunit MCUR1 (Mallilankaraman et al., 2012), and the gate keeper subunits (MICU1, Perocchi et al., 2010; MICU2, Plovanich et al., 2013; MICU3, Patron et al., 2019). The helix-loop-helix structural domains (EF-hand) of the MICU subunits act as Ca^2+^ sensitive regulators and determine the threshold and the cooperative activation of the transport complex (Debattisti et al., 2019). At the same time, NCLX, a member of the Na/Ca exchanger family, was identified as the protein forming the extrusion mechanism (Palty et al., 2010).

## Mitochondria as modulators of cytosolic calcium

In a number of cell types, the uptake and release of calcium by mitochondria is relevant to the cytosolic calcium homeostasis and to cell functions. In presynaptic terminals, free Ca^2+^ concentration is a major determinant of neurotransmitter release (see for a review Schneggenburger and Neher, 2005). Calcium channels and transporters on the plasma membrane as well as on the ER membrane modulate free Ca^2+^ concentration in relation to electrical activity (see for recent reviews Dolphin, 2021; and Broussard and Petreanu, 2021). Mitochondrial uptake contributes to limit the increase of cytosolic calcium and of neurotransmitter release during physiological repetitive electrical activity in presynaptic terminals of lizard motor nerves (David et al., 1998). Consistently, the release of calcium from mitochondria via NCLX counteracts the decline of the post-tetanic release of neurotransmitter in mouse neuromuscular junction (García-Chacón et al., 2006). Likewise, astrocyte cytosolic excitability was found to be enhanced when mitochondrial calcium uptake was inhibited (Boitier et al., 1999). A different, but not less intriguing, role was found for the mitochondria in acinar pancreatic cells (Tinel et al., 1999). Mitochondria, placed around the secretory pole, take up calcium and inhibit diffusion from the primary release sites in the granule-rich area in the apical part of the cell to the basal part containing the nucleus. If mitochondria are depolarized with CCCP (Carbonyl cyanide *m*-chlorophenyl hydrazone), the mitochondrial barrier disappears and a global rise in the cytosolic calcium concentration occurs. Accordingly, in chromaffin cells, calcium uptake by mitochondria, localized at the hotspot close to endoplasmic reticulum and voltage-dependent calcium channel of the plasma membrane, modulates local free (Ca^2+^) and catecholamine secretion (Montero et al., 2000).

Cardiomyocytes are likely the cells with the richest wealth of mitochondria, which cover ∼30% of the cell volume. Thus, the question arises as to whether any flux of Ca^2+^ into the mitochondria may impact the cytosolic Ca^2+^ transient and therefore cardiac contractility. The direct comparison of the cytosolic Ca^2+^ transients in the presence of the MCU inhibitor, Ru360, and in control conditions showed that the amplitude was unchanged. This demonstrated that the calcium taken up by the mitochondria does not modify cytosolic calcium transients (Lu et al., 2013). Similar conclusions based on experimental and computational analysis were reached by Boyman et al. (2014). In contrast, a study on neonatal cardiomyocytes found that knocking down the MCU with siRNA causes a 50% increase of the amplitude of the cytosolic Ca^2+^ transient (Drago et al., 2012). This finding would lead to the conclusion that mitochondria can buffer and modulate the cytosolic calcium transient. As noted by Eisner et al. (2017), there are other important players in calcium flux balance in cardiomyocytes. Actually, the increased amplitude of the calcium transient might be compensated by an increased efflux from the cell, possibly via NCX.

Genetic ablation of MCU either with constitutive knockout (KO; Holmström et al., 2015) or with conditional inducible KO (Kwong et al., 2015; Luongo et al., 2015) did not result in appreciable changes of cytosolic Ca^2+^ concentrations at rest or during electrical stimulation of cardiomyocytes. The ability of mitochondria to quickly take up Ca^2+^ was impaired, while the activity of a still undefined slow uptake mechanism was likely present. Importantly, the conditional inducible KO of NCLX (Luongo et al., 2017), which was lethal in adult mice via calcium overload and permeability transition, was tolerated when started in newborn mice and did not cause any change in cytosolic calcium. Overexpression of NCLX (Luongo et al., 2017) increased the rate of Ca^2+^ efflux from mitochondria but did not alter the cytosolic Ca^2+^ levels. Thus, the presently available evidence rules out a mitochondrial modulation of the cytosolic calcium transients in cardiomyocytes, and identifies the main physiological role of Ca^2+^ uptake in metabolic ATP supply, particularly relevant under stress conditions (Luongo et al., 2015, isoproterenol; Kwong et al., 2015, isoproterenol and treadmill).

## Mitochondria in skeletal muscle fibers

**Figure 1.**
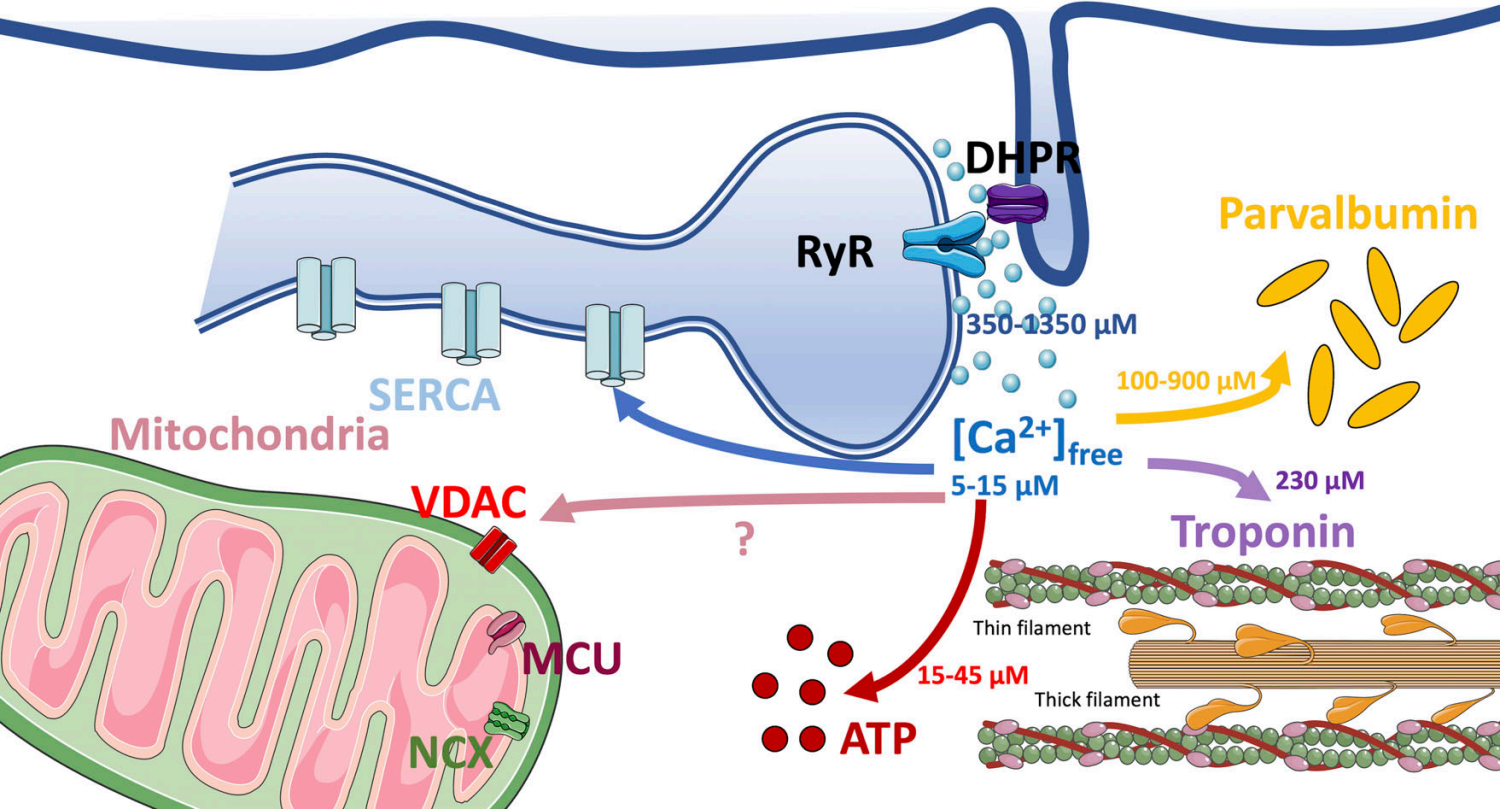
**Schematic representation of the Ca**^**2+**^ **fluxes during excitation–contraction coupling in mammalian fast skeletal muscle fibers.** Depolarization of the transverse tubule triggers Ca^2+^ release from the SR via the contact between DHPR and RYR. The rise in myoplasmic free [Ca^2+^] is followed by interactions with cytosolic Ca^2+^ buffers: PVA, troponin (located on the thin filaments), ATP, and with mitochondrial MCU complex. SERCA Ca^2+^ pump in the SR membranes removes all Ca^2+^ released. Indications of the amount of Ca^2+^ involved in each flux are given in μM (or μmoles/liter fiber volume) and are derived from Baylor and Hollingworth, (2007) and Baylor and Hollingworth (2012).

The remarkable development of the knowledge of the functional relevance of mitochondrial calcium uptake only marginally touched adult skeletal muscles. There was no doubt that mitochondrial calcium uptake played an essential role in the fast activation of the citric acid cycle dehydrogenases and ATP synthesis and, moreover, a role of calcium in permeability transition and apoptosis was widely accepted. After some early debate (see Carafoli et al., 1969), a contribution of the mitochondria to the intracellular calcium homeostasis of adult skeletal muscle fibers was generally excluded for two reasons: (i) the oscillations of cytosolic calcium during the contraction–relaxation cycles are very fast and (ii) the amounts released and taken up by SR are large. In contrast, the initial determinations of mitochondrial calcium uptake in skeletal muscles showed a low uptake rate and a modest calcium storage capability (see below). In accordance with this view, SR acquired the role of central player in releasing to and removing calcium from the cytosol and thus controlling the contraction via troponin binding. The discovery of the RyR-DHPR interactions in the calcium release units (CRUs) provided a beautiful model of fine regulation of Ca^2+^ release in relation to sarcolemma electrical activity (Franzini-Armstrong, 1984; Block et al., 1988). An accepted view of the intrafiber Ca^2+^ fluxes following a release event from SR is illustrated in Fig. 1. The sarcolemma depolarization is followed by a release via RyR estimated at 340–350 μmoles/L_fiber_ (Baylor and Hollingworth, 2007; Baylor and Hollingworth, 2012; Holash and MacIntosh, 2019; Rincón et al., 2021) for a twitch triggered by a single pulse, >550 μmoles/L_fiber_ for a train of five pulses at 67 Hz (Baylor and Hollingworth, 2007; Baylor and Hollingworth, 2012; Rincón et al., 2021) and 1,350 μmoles/L_fiber_ with a 400-ms voltage clamp at +30 mV (Manno et al., 2013). The amount bound by troponin C fast isoform with two calcium-binding sites can reach 240 μmoles/L_fiber_, while parvalbumin (PVA) with a concentration of calcium binding site of 1,260 μmoles/L_fiber_ can bind more than 300 μmoles/L_fiber_ (Baylor and Hollingworth, 2007) and other minor cytosolic buffers as ATP can bind between 5 and 30 μmoles/L_fiber_ (Baylor and Hollingworth, 1998). Then, SERCA is able to bring back to the SR all Ca^2+^ released. Thus, what is left for mitochondria seems irrelevant.

An early attempt to measure the amount taken up by mitochondria and their contribution to relaxation was carried out by Sembrowich and coworkers (Sembrowich et al., 1985), who determined the kinetics and the amplitude of calcium uptake by mitochondria isolated from slow and fast muscle fibers (see Table 1) and reached the conclusion that mitochondria might partially contribute to remove calcium from cytosol in slow fibers, but definitely not in fast fibers. The determination of calcium content and distribution in muscle fibers supported the view that the amount stored in the mitochondria was negligible. It is worth noting, however, that the measurements were performed in static and resting conditions, likely overlooking the calcium redistribution following a release from SR (see Fig. 1). Fryer and Stephenson (1996) with the lysis method concluded that mitochondria calcium content was 0.03–0.04 mmoles/L_fiber_ on a total content of 1.3–1.4 mmoles/L_fiber_ and an SR content of >1 mmol/L_fiber_. Their results were consistent with earlier measurements based on electron probe microanalysis on rapidly frozen cells that yielded a content of 1–4 mmoles/kg dry muscle tissue for the mitochondria vs. 120 for terminal cisternae (Somlyo and Somlyo, 1986). In agreement with those results, the compartmental models of calcium dynamics of Cannel and Allen (1984) and of Baylor and Hollingworth (2007) explicitly excluded the contribution of mitochondria to calcium homeostasis. Baylor and Hollingworth (2007) specify that the transport rate of calcium in the mitochondria is only 0.1% of the rate of calcium release from SR (based on data of Sembrowich et al., 1985). More recently, similar conclusions were reached by measuring calcium content of skeletal muscle with an elegant approach based on Ca-dependent UV absorbance spectra of the Ca^2+^ chelator 1,2-bis(*o*-aminophenoxy)ethane-*N*,*N*,*N′*,*N′*-tetraacetic acid (Lamboley et al., 2015). However, the calcium content in muscles lacking the major SR buffer calsequestrin was found to be higher than expected, suggesting the presence of an alternative site of calcium accumulation. Actually, the recent determination carried out by Lamboley et al. (2021) and coworkers showed an increased total mitochondrial calcium content in muscle fibers with SR depletion due to calsequestrin ablation. This result is in partial agreement with previous observations based on mitochondria-targeted cameleon showing a higher free Ca^2+^ concentration in the mitochondrial matrix of calsequestrin KO muscle fibers (Scorzeto et al., 2013).

In the first decade of the new century, the free Ca^2+^ indicators were applied also in living adult muscle fibers and opened a new perspective, after some initial doubtful results. Lännergren et al. (2001) using Rhod2 reported calcium accumulation in mitochondria during prolonged contractile activity in *Xenopus* fibers but not in mouse fast flexor digitorum brevis (FDB) fibers. 2 yr later, Bruton et al. (2003) measuring cytosolic calcium with Indo-1 and mitochondrial calcium with Rhod2 in mouse soleus and EDL fibers observed, after fatiguing stimulation, an increase of mitochondrial matrix calcium in both muscles. The absence of mitochondrial calcium uptake during tetanic stimulation was confirmed on FDB fibers loaded with Rhod2 (Aydin et al., 2009). In contrast, cameleon probes targeted to mitochondria produced clear-cut evidence of transient increases of mitochondrial Ca^2+^ concentration in electrically stimulated murine muscles with consistent results in tibialis anterior fibers in situ (Rudolf et al., 2004) and in isolated FDB fibers (Scorzeto et al., 2013). The matrix-free Ca^2+^ concentration follows closely cytosolic-free Ca^2+^ variations, even during a single twitch, although with a somewhat different kinetics. A fast rise phase was followed by a slow decrease, and this allowed the integration of the signals coming from repeated stimulations/action potentials. Importantly, the time scale of the metabolic activation triggered by calcium is much faster compared to the activation induced by ADP and creatine which implies energy depletion. Thus, calcium-activation could be interpreted as a feed-forward regulation, while ADP activation appeared as a feed-back control.

The link between calcium and permeability transition of the IMM in adult skeletal muscle fibers found support in the studies of a dystrophic mouse model where collagen VI synthesis was prevented by genetic ablation of the *Col6a1* gene. The analysis of the pathogenic mechanisms pointed to a calcium-mediated dysfunction of mitochondria and SR leading to the opening of the mitochondrial permeability transition pore and apoptosis (Irwin et al., 2003; Angelin et al., 2008).

Recently, additional roles for mitochondrial calcium in skeletal muscle fibers were proposed. The manipulation of MCU expression via plasmidic transfection revealed the regulation of muscle fiber size as a new and unexpected function of calcium uptake (Mammucari et al., 2015). Mitochondrial calcium uptake via MCU was positively correlated with the size of muscle fibers, since an increase in fiber size followed MCU overexpression and a decrease in size accompanied MCU silencing. Moreover, a role of mitochondria in membrane repair has been described (Horn et al., 2017). Mitochondria take up Ca^2+^ entering through the membrane injury and activate ROS production, which stimulates F-actin polymerization, via RhoA.

## Mitochondria as potential modulators of cytosolic calcium in skeletal muscle fibers: Experimental evidence

In the picture outlined in the above sections, a question remains unanswered: can mitochondria control or regulate cytosolic calcium in skeletal muscle fibers, just as they do in several cell types, or are irrelevant as in cardiomyocytes? Can they modify amplitude or kinetics of calcium transients and, therefore, contractile responses? The evidence and the reasoning reported in the previous section play against this possibility. In fast fibers, likely due to the low storage capacity and the slow kinetics of the calcium uptake, the fraction of the calcium released from SR which can be taken up by mitochondria seems minimal. It is definitely sufficient to activate metabolism but not to compete with other calcium binding proteins as troponin C, PVA, and SERCA. Thus, as mentioned above, the mitochondrial contribution to the shaping of cytosolic transient has been dismissed in the modelling of intrafiber calcium dynamics (Cannell and Allen, 1984; Baylor and Hollingworth, 2007; and also in more recent models, see Barclay and Launikonis, 2021). In contrast to this view, however, in the last two decades some evidence emerged suggesting that mitochondria can effectively modulate cytosolic Ca^2+^ and performance in skeletal muscle fibers (see Table 2). Gillis (1997) showed that inhibition of mitochondrial calcium uptake with Ruthenium Red slowed the relaxation process in mitochondria-rich slow skeletal muscles, while in glycolytic fast muscles there was virtually no effect. The experiments were carried out on mechanically skinned muscle fibers and a calcium transient was not recorded. Attempts to assess the contribution of mitochondria in shaping the cytosolic Ca^2+^ transient were carried out by impairing the ability to take up Ca^2+^ by depolarizing the IMM. Beside the expected surge of cytosolic Ca^2+^ concentration, the exposure of isolated FDB fibers to 2 μM FCCP (carbonyl cyanide-*p-* trifluoromethoxy-phenylhydrazone) decreased the amplitude of the cytosolic transient induced by a short voltage clamp with a slowing of the decay phase (Weiss et al., 2010) or by a short tetanic stimulation, without any delay of the relaxation phase (Caputo and Bolaños, 2008). Surprisingly, Andrade and coworkers (Andrade et al., 2005 found that, in extraocular muscle, mitochondria depolarization with CCCP (10 μM) was accompanied by an increment of ∼20% of the cytosolic transients induced by repetitive electrical stimulation (50–300 Hz) and attributed this effect to the peculiar abundance of mitochondria in extraocular muscle, ∼15% of fiber volume.

**Table 2.** **Experimental results showing the impact of altered mitochondrial Ca**^**2+**^ **uptake on cytosolic Ca**^**2+**^ **concentration**.

Reference	Experimental model and protocol	Cytosolic transient amplitude	Cytosolic transient duration		Mitochondrial transient amplitude
Gillis, 1997	Mechanically skinned fibers Rhutenium Red	nd	delayed relaxation half time +176%		nd
Caputo and Bolanos., 2008	FDB fibers Electrical stimulation FCCP 2 μM	decreased −46%	no change		nd
Weiss et al., 2010	FDB fibers Electrical stimulation FCCP 2.5 μM	decreased −55%	prolonged −37% (rate constant)		nd
Yi et al., 2011	FDB fibers G93A SOD1 mutation Voltage clamp	increased +15.6%	no change		decreased −14.9%
Andrade et al., 2005	Intact EO muscles Electrical stimulation CCCP 10 μM	increased +72%	nd		nd
Ainbinder et al., 2015	FDB fibers Electrical stimulation Mfn2 downregulation	increased twitch +10% Tetanus + 30%	no change		decreased −30%
Eisner et al., 2014	FDB fibers Electrical stimulation Mfn1 KO and downreg	Increased decay with repeated stimulation	nd		decreased uptake
Mammucari et al., 2015	FDB fibers–caffeine MCU silencing	no change	nd		decreased −45%
	FDB fibers–caffeine MCU upregulation	decreased −10%	nd		increased +76%
Gherardi et al. 2019	FDB fibers–caffeine MCU–constitutive KO	decreased −10%	nd		decreased to 0
Favaro et al., 2019	FDB fibers Electrical stimulation DRP1-KO (MCU upregulation)	decreased −9%	nd		increased +14.2%
Debattisti et al., 2019	FDB fibers Electrical stimulation MICU1 KO	trend to decrease	no change		decreased −30%
Butera et al., 2021	FDB fibers–caffeine PVA KO and MCU down regulation	increased +36%	nd		decreased −28%

The percent variations are in some cases directly reported in the papers and in some cases recalculated from histograms or tables. nd, not determined.

Further evidence in favor of a mitochondrial modulation of the cytosolic Ca^2+^ transients came from the analysis of intact skeletal muscles fibers isolated from mice carrying A93G mutation of SOD1, a model of hereditary amyotrophic lateral sclerosis (Yi et al., 2011). Those fibers displayed segmental impairment of mitochondrial function and the amplitude of the cytosolic Ca^2+^ transients induced by voltage clamp was ∼15% greater in the segments with dysfunctional mitochondria than in the healthy segments.

The mitochondrial protein Mitofusin 1 (Mfn1) and Mitofusin 2 (Mfn2) are key players in mitochondrial fusion as well as in the contacts with SR which are instrumental to effective mitochondrial Ca^2+^ uptake. Selective silencing indicates that Mfn1 has a more important role than Mfn2 in mitochondrial connectivity and fusion (Eisner et al., 2014). While acute downregulation of Mfn1 did not alter cytosolic calcium transients, long-term downregulation, which can be induced by chronic ethanol consumption, caused an instability of the Ca^2+^ release-uptake cycles, detectable as irregular amplitude of the cytosolic transients during tetanic stimulation (Eisner et al., 2014). The downregulation of Mfn2 via shRNA transfection (Ainbinder et al., 2015) caused fragmentation and displacement of mitochondria. The number of mitochondria close to the CRU was reduced by 60%. Compared to control fibers, the mitochondrial Ca^2+^ transient during repeated electrical stimulation (0.5 s tetani at 100 Hz) was reduced, while the cytosolic transient amplitude was increased. It is worth noting that MCU expression was unchanged but mitochondria were partially depolarized.

The identification of the gene coding for MCU (De Stefani et al., 2011) allowed the direct manipulation of MCU expression by plasmid transfection in murine FDB muscles. MCU silencing with shRNA was associated with a decrease of mitochondrial matrix calcium concentration without changes of cytosolic concentration at rest or during a caffeine-induced calcium release. In contrast, MCU overexpression increased the intramitochondrial calcium concentration, accompanied by a modest reduction of the cytosolic calcium level following caffeine-induced calcium release (Mammucari et al., 2015).

In FDB muscle fibers with constitutive MCU ablation, mitochondria were unable to take up Ca^2+^ during the cytosolic Ca^2+^ wave induced by caffeine administration. Surprisingly, the amplitude of the cytosolic Ca^2+^ wave was somewhat lower in MCU-KO than in WT fibers, suggesting a negative effect of chronic MCU deletion on global calcium homeostasis (Gherardi et al., 2019). Basal cytosolic Ca^2+^ levels were unaffected by MCU gene deletion.

The relevance of MCU, and therefore of the mitochondrial calcium uptake, to cytosolic calcium was confirmed by the analysis of a mouse model of altered mitochondrial dynamics (Favaro et al., 2019). Muscle-specific ablation of the pro-fission dynamin related protein 1 (DRP1) by conditional KO-induced muscle wasting and weakness. In DRP1-KO muscle fibers, mitochondria were bigger and morphologically and functionally abnormal. MCU protein expression was increased via miRNA1 downregulation, and this enhanced Ca^2+^ uptake by mitochondria. Cytosolic transients induced by electrical stimulation or caffeine administration were reduced in amplitude, while the mitochondrial transients were magnified. The free Ca^2+^ concentrations in the SR and the calsequestrin levels were unchanged, in spite of some morphological alterations and a decreased density of the CRUs.

A recent study (Butera et al., 2021) on FDB muscle fibers of mice carrying a null mutation of *Pvalb* gene showed that the absence of a major cytoplasmic buffer (PVA) was not accompanied by alterations of the amplitude of cytosolic Ca^2+^ transient induced either by caffeine or electrical stimulation. However, when MCU expression was silenced by plasmid shRNA transfection, the amplitude of cytosolic Ca^2+^ transients was increased in PVA KO but not in WT fibers, thus suggesting that mitochondrial Ca^2+^ uptake can compensate for PVA absence, to some extent. This possibility is even more relevant, considering that PVA in fast skeletal muscles is supposed to absorb up to several hundreds of µmoles/L_fiber_ during a twitch or a train of stimuli, as stated above (see Fig. 1).

The regulatory subunits MICU1 and MICU2 of the Ca^2+^ uptake complex play an important role in gate-keeping and cooperative activation of MCU complex (Debattisti et al., 2019). Consistently, the deletion of MICU1 gene in FDB muscle fibers was followed by a lowering of the threshold for Ca^2+^ uptake and a reduced cooperativity. Free Ca^2+^ concentration in the mitochondrial matrix showed a higher basal level. Electrical stimulation, twitch and tetanus, induced mitochondrial transients of reduced amplitude, likely due to the loss of cooperativity of the MCU complex, while the cytosolic transients showed a tendency to smaller amplitude (Debattisti et al., 2019).

Taken together, the evidence reported above are definitely in favor of a contribution of mitochondrial Ca^2+^ uptake to the modulation of cytosolic calcium transients, under specific circumstances. There are, however, some contradictory aspects and some possible alternative explanations that deserve attention. In muscle fibers poisoned with FCCP, the reduction of transient amplitude might be due to a Ca^2+^-dependent inactivation of Ca^2+^ release from SR or to a complete disruption of excitation–contraction coupling mechanisms (Caputo and Bolaños, 2008) following the large release of Ca^2+^ induced by mitochondrial depolarization (Vajda et al., 2009). However, the increased amplitude of the cytosolic Ca^2+^ transient evoked by voltage clamp with a parallel reduction of mitochondrial matrix transient observed in SOD1-A93G muscle fibers with mitochondrial dysfunction (Yi et al., 2011) can be correctly interpreted as a demonstration that mitochondria modulate cytosolic Ca^2+^ transient by buffering about 20% of the amount released by SR. The genetic (KO, downregulation, and overexpression) or pharmacological (Ruthenium Red) manipulation of MCU provides more refined approach compared to IMM depolarization. The prolongation of the relaxation phase observed by Gillis following inhibition of MCU with Ruthenium Red (Gillis, 1997) might be caused by the persistence of higher levels of Ca^2+^ in the cytosol. Interestingly, this effect was observed in slow but not in fast muscle fibers. Actually, downregulation of MCU with shRNA transfection does not produce any effect on cytosolic Ca^2+^ concentration in fast FDB muscle fibers (Mammucari et al., 2015) in spite of a reduction of the Ca^2+^ levels in the mitochondrial matrix. However, when combined with PVA ablation, thus a condition resembling slow fibers, MCU downregulation is accompanied by an increased amplitude of the cytosolic Ca^2+^ transients (Butera et al., 2021). In fast fibers, PVA can fulfil its task of main cytosolic buffer and dampen the possible increase in cytosolic Ca^2+^ due to reduced mitochondrial uptake (see also below the simulated changes in uptake rate). When MCU expression is upregulated either via direct plasmid transfection (Mammucari et al., 2015) or via a more complex mechanism based on miRNA1 (Favaro et al., 2019), the mitochondrial transient is amplified, while the cytosolic transient is somewhat reduced. This suggests that the number of MCU channels available or their density on the IMM is a limiting factor for the ability of mitochondria to buffer cytosolic calcium.

The variations of mitochondrial calcium can be also considered in the perspective of a bidirectional mitochondria-SR signaling (Dirksen, 2009; Booth et al., 2021), i.e., the variations of mitochondrial calcium could influence directly the SR function and this, in turn, modulated the cytosolic transients. While calcium is the player of the orthograde signaling from SR to mitochondria, ROS can convey the retrograde signaling and act on RyR (Sun et al., 2013), on IP_3_R (Booth et al., 2021), inducing leakage and increased permeability, and on SERCA, depressing calcium transport rate (Chernorudskiy and Zito, 2017). This can lead to a multiplicative effect on the levels of cytosolic calcium in specific conditions, e.g., RyR mutations causing increased leakage during a malignant hyperthermia crisis (Canato et al., 2019). Besides ROS, among molecular signals sent by mitochondria to SR, BCL-2 is known to display an inhibitory effect on RyR permeability (Pinton and Rizzuto, 2006; Ivanova et al., 2020). A release of BCL-2 following an increase in mitochondrial calcium might be speculatively considered as negative feed-back loop. On the whole, we cannot exclude that the changes in mitochondrial uptake are linked to changes in cytosolic Ca^2+^ transients by variations in SR release/uptake induced by signals originated in the mitochondria.

## Mitochondria as potential modulators of cytosolic calcium in skeletal muscle fibers: Quantitative constraints

Moving to the quantitative level, the evidence discussed above and summarized in Table 2 suggest that mitochondria are able to remove up to 20% of the calcium released by SR (Yi et al., 2011), in murine fast fibers. If the Ca^2+^ released from SR is estimated between 350 (single pulse) and 1,200 (voltage clamp) μmoles/L_fiber_ (see above and Fig. 1), 70–240 μmoles/L_fiber_ are expected to be taken up by the mitochondria during electrical stimulation. Since mitochondria volume in fast murine fibers is ∼5% of the fiber volume, the intramitochondrial concentration should rise to 1.4–4.8 mM (or mmoles/L_mito_), which is >1,000 times above the free Ca^2+^ concentration actually measured in mitochondrial matrix with various calcium probes (0.1 and 2 μM, see above). This points to a very high buffer capacity of the mitochondrial matrix, compatible with a high “total calcium/free calcium” ratio.

While several studies (see above; Somlyo and Somlyo, 1986; Fryer and Stephenson, 1996; Lamboley et al., 2015) ruled out the presence of a significant fraction of total calcium in the mitochondria of skeletal muscles, Madsen et al. (1996) and coworkers measured a calcium concentration of 11–15 nmol/mg protein in mitochondria isolated from human muscle with atomic absorption spectrometry. Such concentration, assuming that 1 mg corresponds to 2.63 μl (Schwerzmann et al., 1989; see also caption of Table 1), would lead to 4.2–5.7 mmoles/L_mito_, i.e., about 2,000–10,000 times the free Ca^2+^ concentration in the matrix. Although very high, such value is compatible with the determinations in liver, brain, and heart (see Chalmers and Nicholls, 2003; Bazil et al., 2013) and requires a high buffering power. At the best of our knowledge, the few attempts to simulate the amount of calcium accumulated in the mitochondria during contractile activity of fast skeletal muscle fibers have assumed a much lower buffering power, ∼200:1 (Marcucci et al., 2018; Rincón et al., 2021), similar to the value proposed by Wust et al. (2017) for cardiomyocytes.

The recent paper by Lamboley et al. (2021) and coworkers has provided the first direct measurement of bound-to-free calcium ratio in mitochondria in FDB muscle fibers, the most used model of intact adult skeletal muscle fibers ex vivo. The total amount of calcium was measured by applying the lysis method, designed by Fryer and Stephenson (1996). In the same set of fibers, the determination of free Ca^2+^ concentration in the matrix using Rhod-2 as probe and of mitochondrial volume by morphometry applied to electron microscopy images, provided a measurement of the amount of free Ca^2+^ present and the ratio total/free gave an indication of the potential buffering power. The calculated values range between 4,000 and 10,000, the latter value only in genetically modified muscle fibers. Although these determinations were performed in static conditions, in resting fibers, they demonstrated that skeletal muscle mitochondria have a high capacity to accumulate calcium, keeping low the free Ca^2+^ concentration in the matrix.

The mitochondrial ability to modulate cytosolic Ca^2+^ during a release event from SR, which lasts seconds or fraction of seconds, is not only matter of amount, but also of speed in taking up, storing, and releasing calcium. The variations of mitochondrial free Ca^2+^ (d [Ca^2+^]_mito_/dt) detectable with appropriate Ca^2+^ sensors are dependent not only on the uptake rate (J_in_), but also on the extrusion rate (J_out_) and on the buffering rate (J_b_).

$$\frac{d{\left[{Ca}^{2+}\right]}_{mito}}{dt}=J_{in}–J_{out}-J_{b}\text{,}$$ (1)

where J_b_ = Σ(k_on_·[Ca^2+^]_mito_·[B_tot_ − B_Ca_] – k_off_. [B_Ca_])_I_, with B_tot_ the total amount, B_Ca_ the amount bound to calcium, and k_on_ and k_off_ are the rate constants of the buffer I present in the matrix. Quantitative determinations based on mitochondrial cameleon (Scorzeto et al., 2013) showed that during repetitive electrical stimulation at 60 Hz, free Ca^2+^ concentration in the mitochondrial matrix climbs to 1–2 μM in ∼1 s. Assuming a buffering up to 5,000 times, this means a rate of uptake of 5–10 mmoles/L_mito_/s, or 250–500 μmoles/L_fiber_/s. A rate of 4.1 mmoles/L_fiber_/s has been calculated by Yi et al. (2011) without considering a simultaneous export via NCLX.

As mentioned above, values between 12 and 37 μmoles/L_fiber_/s were determined by Sembrowich et al. (1985) in mitochondria isolated from rat and rabbit muscles. Such values correspond to 0.24 and 0.74 mmoles/L_mito_/s. These values are rather low compared to determinations in similar experimental conditions on mitochondria isolated from liver or heart (see Table 1), which yielded values between 3 and 11 mmoles/L_mito_/s.

Patch clamp in mitoplasts might offered a way to directly measure the uptake current. Mitoplasts, however, lack the outer mitochondrial membrane where VDAC channels allow the entry of Ca^2+^ into the intermembrane space, possibly in a regulated manner (Sander et al., 2021). Mitochondrial calcium uptake rate can be estimated from MCU current expressed in pA/pF, assuming an inner mitochondrial membrane capacitance of 10 mF/m^2^ (Padmaraj et al., 2014) with a total surface of 1,500 m^2^/L_fiber_ (Schwerzmann et al., 1989) to obtain, through the definition of Coulomb and considering two electrical unitary charges per Ca^2+^ ion, a conversion factor of

$$\Theta_{fiber}=10\frac{mF}{m^{2}}1,500\frac{m^{2}}{L_{fiber}}6.2\cdot 10^{15}\frac{e}{mA\cdot s}\frac{1}{2\cdot 6.02\cdot 10^{23}}\frac{mol}{e}=0.077\frac{mmol}{L_{fiber}s}\frac{pF}{pA}\text{.}$$

The MCU current of 58 pA/pF, a value higher than in any other tissue analyzed, was measured by Fieni et al. (2012) in mitoplasts from skeletal muscles, in the presence of 100 μM Ca^2+^ and Ψ = −160 mV. The above algorithm yields a value of maximal uptake rate of 4.3 mmoles/L_fiber_/s.

As mentioned above, MCU complex has been since long time characterized as a low affinity mechanism, the K_0.5_ ranging between 3 and 90 μM (see Table 1), thus well above any cytosolic Ca^2+^ concentration. The determination on isolated mitochondria places skeletal muscles at the lower end with half maximal rate reached at 2–3 μM (Sembrowich et al., 1985). This condition, however, would not guarantee to reach high uptake rates without the presence of a microdomain with peculiar Ca^2+^ concentration. In muscle fibers, intermyofibrillar mitochondria located close to the CRU can enjoy a relatively favorable condition for calcium uptake. Recent determinations of Ca^2+^ concentrations in the triadic space using a targeted variant of GCaMP6f (Sanchez et al., 2021) suggest that 40 μM are reached during a voltage clamp in FDB adult muscle fibers.

**Figure 2.**
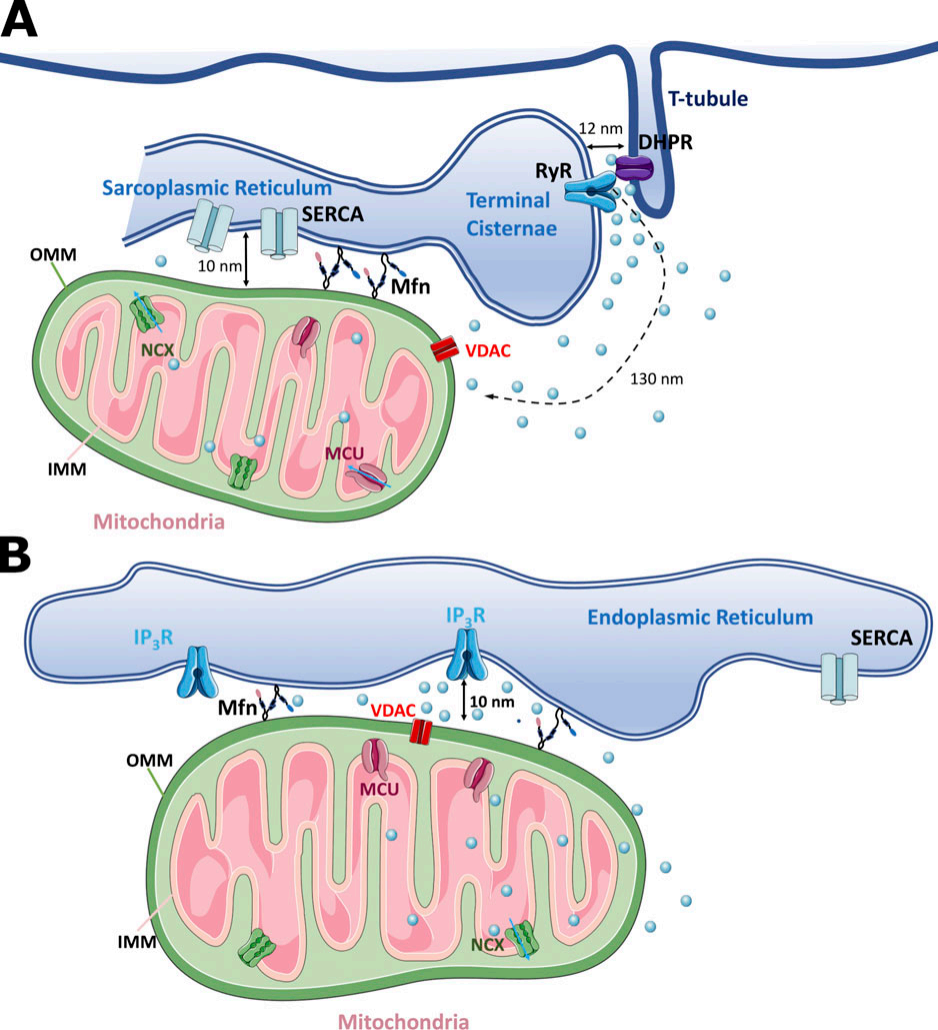
**Schematic representation of the spatial interactions between ER/SR and mitochondria in adult muscle fibers compared to other cell types.** **(A and B) **Note that, in skeletal muscle fibers (A), in spite of the close relation between mitochondria and SR/ER membranes, supported by the presence of mitofusins (Mfn), the distance between MCU and RyR is relatively large (>130 nm), due to the position of RyR on terminal cisternae in front of DHPR on T-tubules. OMM, outer mitochondrial membrane. In contrast, in other cell types (B) the distance between calcium release channels (IP_3_R) and mitochondrial membrane is much shorter (10 nm).

The estimated distance between RyR and MCU in intermyofibrillar mitochondria is worth of comments (see Fig. 2). In skeletal muscle fibers, the distance is not <130 nm due to the peculiar localization of RyR in the junctional space facing and touching DHPR on the T-tubule membrane (Boncompagni et al., 2009; Dirksen, 2009; see Fig. 2 A), while in most cell types the distance between RyR-MCU or IP_3_R-MCU is much shorter (see Fig. 2 B), in the order of few tens nm, and strictly controlled by protein tethers, among them Mfn1 and Mfn2. In spite of the greater distance, the concentration of free Ca^2+^ experienced by the mitochondrial transport system can be predicted to be well above the average cytosolic concentration. Multicompartmental models help to calculate the concentrations, assuming a diffusion rate of calcium equal to 3 × 10^-6^ cm^2^/s. According to the multicompartmental (compartment number: *n* = 18) model proposed by Baylor and Hollingworth (2007), at the sarcomere length of 2.4 µm, the distances between the calcium release compartment or CRU (the outermost) and the two isovolumetric compartments disposed radially towards the center of the myofibrils, are, respectively, 146 and 344 nm. At the distance of 146 nm, similar to the position of a mitochondrion, not considered in the model, the peak free Ca^2+^ concentration is 75% of the concentration at the release site and still four times higher than the cytosol average. Similar calculations, based on the multicompartmental model (*n* = 15) proposed by Holash and MacIntosh (2019) show that moving from the release site to the adjacent compartments either longitudinally or radially at an average distance of 230 nm the peak free Ca^2+^ concentration decreases to 50% compared to the release site and is still 50% higher than the cytosolic average. At variance of the two multicompartmental models considered, the multicompartmental model (*n* = 15) designed by Marcucci et al. (2018) explicitly takes into account the mitochondria and predicts values of free Ca^2+^ concentration in the microdomain two times greater than the average cytosolic values. The relevance of the mitochondria localization close to the CRU finds an experimental support in the studies by Eisner et al. (2014) and Ainbinder et al. (2015) on the impact of downregulation of Mfn1 and Mfn2. Not only the mitochondrial calcium uptake is impaired but also the amplitude of the cytosolic calcium transients is altered.

It is important to underline that, in skeletal muscles, the response of MCU complex to cytosolic Ca^2+^ is enhanced by the expression of a specific isoform of MICU1, called MICU1.1 (Vecellio Reane et al., 2016). The affinity for Ca^2+^ of MICU1.1 is about 10 times greater than that of MICU1: 56 and 130 nM, respectively, for the two EF sites, versus 21.4 and 15.8 μM (see Wang et al., 2014). This allows the opening of the gate at lower cytosolic Ca^2+^ concentration with a significant Ca^2+^ uptake even below the threshold of 300–400 nM.

**Figure 3.**
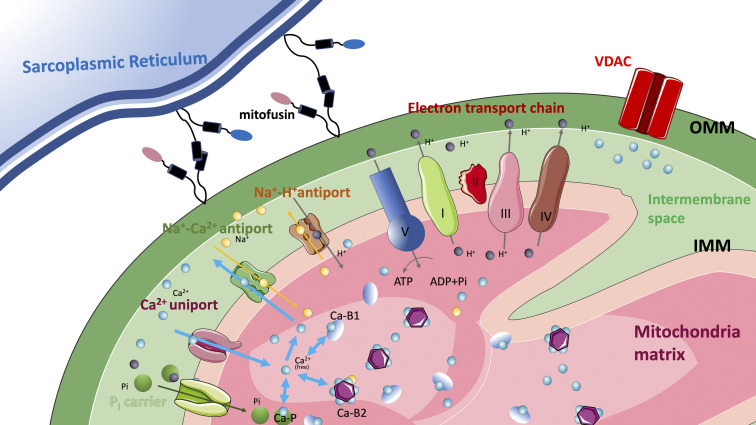
**Schematic representation of the Ca**^**2+**^ **movements through the outer (OMM) and inner (IMM) mitochondrial membrane and of the binding to mitochondrial matrix buffers.** Three main types of calcium buffers are shown: Ca-P represents calcium phosphate complex and B1 and B2 summarize all other possible binding molecules.

The uptake rate, however, is only one of the determinants of ability of mitochondria to accumulate and release Ca^2+^ in a time scale relevant for physiological muscle contraction (see Eq. 1). The kinetics of the mitochondrial Ca^2+^ storage system is not less important. Two types of calcium sequestering systems have been identified in cardiac mitochondria (Bazil et al., 2013; Wei et al., 2012). The first class represents specific Ca^2+^ buffers, while the second class is based on the formation of amorphous calcium phosphate (see Fig. 3). If the Ca^2+^ binding kinetics of the two or possibly more classes of mitochondrial Ca^2+^ binding molecules are comparable to the inward and outward fluxes, then they can act as buffers and their combined activity can account for the powerful ability to accumulate substantial amounts of calcium with minor variation of free Ca^2+^ concentration in the matrix. This represents a precondition to contribute to the modulation of cytosolic Ca^2+^ transients. The available model estimations adopt the following parameters: Marcucci et al., 2018: k_on_ = 0.8 μM^−1^ s^−1^, k_off_ 0.19 s^−1^, k_d_ = 0.24 μM; Rincón et al., 2021: k_on_ = 0.6 μM^−1^ s^−1^, k_off_ 0.156 s^−1^, k_d_ = 0.26 μM. For comparison, the k_d_ calculated in Bazil et al. (2013) for cardiac mitochondria are 2 and 1.7 μM, respectively, for the two classes of buffers, while the kinetic parameters are assumed to be fast enough to rapidly reach the equilibrium, at least in the time scale of their experimental data, i.e., in the order of several minutes.

**Figure 4.**
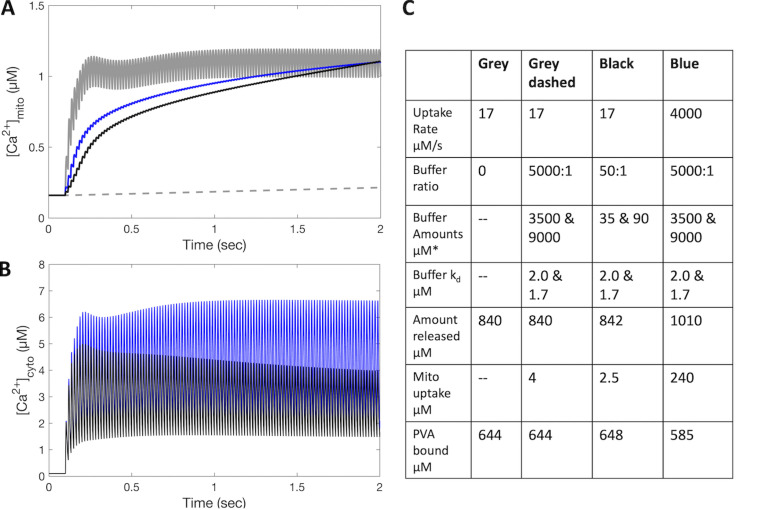
**Model simulation of the relative impact of uptake rate and buffering rate (Eq. 1: J**_**in**_ **and J**_**b**_**) on the mitochondrial Ca**^**2+**^ **transient and on the cytosolic Ca**^**2+**^ **transient. (A–C)** The transients in response to 2-s 60-Hz electrical stimulation are simulated with the model described by Marcucci et al. (2018). The relevant parameters are shown in the table (C). Four cases are considered with different uptake rates and different buffer properties. The two buffers and their k_d_ derive from Bazil et al. (2013). The grey lines in A show the combination of an uptake rate of 17 μmoles/L_fiber_/s (=350 μmoles/L_mito_/s; Sembrovich et al., 1985) with no buffer or high buffer (see Lamboley et al., 2021): in the former case the mitochondrial transient follows the cytosolic transient, in the latter case is canceled by the fast buffering. The blue and the black lines show the combination of low J_in_ with low buffer or high J_in_ with high buffer power. The difference is small in the mitochondrial transient but clearly visible on cytosolic transient, in spite a partial compensation by increased binding to parvalbumin. Troponin C, not shown in the figure, also binds more Ca^2+^ in the blue case, thus contractile response is enhanced. Molar concentrations (μM) refer to L_fiber_, except the buffer amounts (μM*), which are expressed in L_mito_.

A model simulation (Fig. 4) of the mitochondrial free Ca^2+^ transients as determined with Ca^2+^ indicators allows some speculations on the relative impact of the three main components of Eq 1. The cytosolic and the mitochondrial transients during a tetanic stimulation (2 s at 60 Hz) are simulated assuming a combination of different uptake rate (J_in_; Eq 1) and buffer-binding rate (J_b_). The impact of two extreme uptake rates, the low rate estimated by Sembrovich et al. (1985), and the high rate similar to that reported by Yi et al. (2011) (4 mmol/L_fiber_/s), are analyzed in different buffering conditions, modulating buffering power through its amount. Buffer parameters are based on Bazil et al. (2013), with the association and dissociation rate constants sufficiently high and the proposed k_d_ values. As can be seen in Fig. 4, the combination of low J_in_ and low J_b_ (blue line) and the combination of high J_in_ and high J_b_ (black line) predict a similar mitochondrial transient, but different cytosolic transient. The impact on cytosolic transient, which will be accompanied by proportional troponin C binding and contractile response, is then partially blunted by compensatory buffering of PVA.

The experimental mitochondrial free Ca^2+^ transients show a pronounced asymmetry with a decay phase much slower than the rising phase both in cardiomyocytes (Lu et al., 2013; Wüst et al., 2017) and skeletal muscle fibers (Rudolf et al., 2004; Scorzeto et al., 2013). The decay phase has been characterized with a rate constant of 0.2 s^−1^ (Scorzeto et al., 2013). The slow decay can be due to a slow release from the buffer keeping free Ca^2+^ high, but only if it is associated to a similar slow kinetics of the NCLX. This brings to consider the third potential limiting factor, i.e., the rate of extrusion from mitochondria via NCLX. A much faster calcium extrusion would lead to low levels of free Ca^2+^, even in the case that the total mitochondrial calcium required longer time to be fully extruded due to binding to matrix or IMM molecules, and this is not observed experimentally. This is even more remarkable considering that, after the end of the electrical stimulation, the cytosolic Ca^2+^ decreases abruptly (see for example Canato et al., 2019), and therefore Ca^2+^ entry through the MCU complex rapidly drops to basal levels, while the Ca^2+^ extrusion should be kept high by the high intramitochondrial Ca^2+^. This supports the view that the calcium sequestration system inside the mitochondria acts as a buffer. An extreme, well-documented case is the slow extrusion of Ca^2+^ from mitochondria in motor nerve terminals, which can allow a prolonged neurotransmitter release (García-Chacón et al., 2006). To summarize, the values of all three terms in the right-hand side of Eq. 1 are important for the understanding of the role of the mitochondria in modulating the cytosolic Ca^2+^ during physiological contractions. More experimental data is needed in this direction, to clarify not only how much Ca^2+^ can be taken up and how fast, but also how is stored and how the extrusion contributes.

## Conclusion

In conclusion, a new perspective seems to emerge in the multifaceted relations between calcium and mitochondria of skeletal muscle fibers. In addition to the regulation of Krebs cycle and ATP generation (Denton et al., 1978), to the triggering of PTP opening and cellular death (Fontaine et al., 1998), and to the intracellular signaling for fiber size control (Mammucari et al., 2015) or membrane repair (Horn et al., 2017), mitochondria appear as potential players of the cytosolic calcium homeostasis and, through this, also modulators of the contractile response. In our opinion, however, it is still disputable whether this control is relevant in physiological conditions, i.e., when fibers accomplish their usual contractile tasks, or represents a reserve mechanism to be utilized in specific conditions, for example, the lack of PVA (Butera et al., 2021), or to be activated when the capacity of calcium uptake is enhanced by overexpression of MCU (Mammucari et al., 2015).

## References

[bib1] Ainbinder, A., S. Boncompagni, F. Protasi, and R.T. Dirksen. 2015. Role of Mitofusin-2 in mitochondrial localization and calcium uptake in skeletal muscle. Cell Calcium. 57:14–24. 10.1016/j.ceca.2014.11.002 25477138 PMC4300280

[bib2] Andrade, F.H., C.A. McMullen, and R.E. Rumbaut. 2005. Mitochondria are fast Ca^2+^ sinks in rat extraocular muscles: A novel regulatory influence on contractile function and metabolism. Invest. Ophthalmol. Vis. Sci. 46:4541–4547. 10.1167/iovs.05-0809 16303946

[bib3] Angelin, A., P. Bonaldo, and P. Bernardi. 2008. Altered threshold of the mitochondrial permeability transition pore in Ullrich congenital muscular dystrophy. Biochim. Biophys. Acta. 1777:893–896. 10.1016/j.bbabio.2008.03.026 18435905

[bib4] Aracena-Parks, P., S.A. Goonasekera, C.P. Gilman, R.T. Dirksen, C. Hidalgo, and S.L. Hamilton. 2006. Identification of cysteines involved in S-nitrosylation, S-glutathionylation, and oxidation to disulfides in ryanodine receptor type 1. J. Biol. Chem. 281:40354–40368. 10.1074/jbc.M600876200 17071618

[bib5] Arnaudeau, S., W.L. Kelley, J.V. Walsh, and N. Demaurex. 2001. Mitochondria recycle Ca(2+) to the endoplasmic reticulum and prevent the depletion of neighboring endoplasmic reticulum regions. J. Biol. Chem. 276:29430–29439. 10.1074/jbc.M103274200 11358971

[bib6] Aydin, J., D.C. Andersson, S.L. Hänninen, A. Wredenberg, P. Tavi, C.B. Park, N.-G. Larsson, J.D. Bruton, and H. Westerblad. 2009. Increased mitochondrial Ca^2+^ and decreased sarcoplasmic reticulum Ca^2+^ in mitochondrial myopathy. Hum. Mol. Genet. 18:278–288. 10.1093/hmg/ddn355 18945718

[bib7] Babcock, D.F., J. Herrington, P.C. Goodwin, Y.B. Park, and B. Hille. 1997. Mitochondrial participation in the intracellular Ca^2+^ network. J. Cell. Biol. 136:833–844. 10.1083/jcb.136.4.833 9049249 PMC2132502

[bib8] Barclay, C.J., and B.S. Launikonis. 2021. Components of activation heat in skeletal muscle. J. Muscle Res. Cell Motil. 42:1–16. 10.1007/s10974-019-09547-5 31346851

[bib9] Baughman, J.M., F. Perocchi, H.S. Girgis, M. Plovanich, C.A. Belcher-Timme, Y. Sancak, X.R. Bao, L. Strittmatter, O. Goldberger, R.L. Bogorad, . 2011. Integrative genomics identifies MCU as an essential component of the mitochondrial calcium uniporter. Nature. 476:341–345. 10.1038/nature10234 21685886 PMC3486726

[bib10] Baylor, S.M., and S. Hollingworth. 1998. Model of sarcomeric Ca^2+^ movements, including ATP Ca^2+^ binding and diffusion, during activation of frog skeletal muscle. J. Gen. Physiol. 112:297–316. 10.1085/jgp.112.3.297 9725890 PMC2229419

[bib11] Baylor, S.M., and S. Hollingworth. 2007. Simulation of Ca^2+^ movements within the sarcomere of fast-twitch mouse fibers stimulated by action potentials. J. Gen. Physiol. 130:283–302. 10.1085/jgp.200709827 17724162 PMC2151645

[bib12] Baylor, S.M., and S. Hollingworth. 2012. Intracellular calcium movements during excitation-contraction coupling in mammalian slow-twitch and fast-twitch muscle fibers. J. Gen. Physiol. 139:261–272. 10.1085/jgp.201210773 22450485 PMC3315149

[bib13] Bazil, J.N., C.A. Blomeyer, R.K. Pradhan, A.K.S. Camara, and R.K. Dash. 2013. Modeling the calcium sequestration system in isolated Guinea pig cardiac mitochondria. J. Bioenerg. Biomembr. 45:177–188. 10.1007/s10863-012-9488-2 23180139 PMC3615037

[bib14] Block, B.A., T. Imagawa, K.P. Campbell, and C. Franzini-Armstrong. 1988. Structural evidence for direct interaction between the molecular components of the transverse tubule/sarcoplasmic reticulum junction in skeletal muscle. J. Cell. Biol. 107:2587–2600. 10.1083/jcb.107.6.2587 2849609 PMC2115675

[bib15] Boitier, E., R. Rea, and M.R. Duchen. 1999. Mitochondria exert a negative feedback on the propagation of intracellular Ca^2+^ waves in rat cortical astrocytes. J. Cell. Biol. 145:795–808. 10.1083/jcb.145.4.795 10330407 PMC2133193

[bib16] Boncompagni, S., A.E. Rossi, M. Micaroni, G.V. Beznoussenko, R.S. Polishchuk, R.T. Dirksen, and F. Protasi. 2009. Mitochondria are linked to calcium stores in striated muscle by developmentally regulated tethering structures. Mol. Biol. Cell. 20:1058–1067. 10.1091/mbc.E08-07-0783 19037102 PMC2633377

[bib17] Booth, D.M., P. Várnai, S.K. Joseph, and G. Hajnóczky. 2021. Oxidative bursts of single mitochondria mediate retrograde signaling toward the ER. Mol. Cell. 81:3866–3876.e2. 10.1016/j.molcel.2021.07.014 34352204 PMC8455442

[bib18] Boyman, L., A.C. Chikando, G.S.B. Williams, R.J. Khairallah, S. Kettlewell, C.W. Ward, G.L. Smith, J.P.Y. Kao, and W.J. Lederer. 2014. Calcium movement in cardiac mitochondria. Biophys. J. 107:1289–1301. 10.1016/j.bpj.2014.07.045 25229137 PMC4167535

[bib19] Brierley, G., E. Murer, E. Bachmann, and D.E. Green. 1963. Studies on ion transport. ii. The accumulation of inorganic phosphate and magnesium ions by heart mitochondria. J. Biol. Chem. 238:3482–3489. 10.1016/s0021-9258(18)48693-0 14085406

[bib126] Bragadin, M., T. Pozzan, and G.F. Azzone. 1979. Kinetics of Ca^2+^ carrier in rat liver mitochondria. Biochemistry. 18:5972–5978. 10.1021/bi00593a033 42437

[bib20] Brierley, G.P., E. Bachmann, and D.E. Green. 1962. Active transport of inorganic phosphate and magnesium ions by beef heart mitochondria. Proc. Natl. Acad. Sci. USA. 48:1928–1935. 10.1073/pnas.48.11.1928 14015424 PMC221100

[bib21] Brini, M., F. De Giorgi, M. Murgia, R. Marsault, M.L. Massimino, M. Cantini, R. Rizzuto, and T. Pozzan. 1997. Subcellular analysis of Ca^2+^ homeostasis in primary cultures of skeletal muscle myotubes. Mol. Biol. Cell. 8:129–143. 10.1091/mbc.8.1.129 9017601 PMC276065

[bib22] Brinley, F.J., T. Tiffert, and A. Scarpa. 1978. Mitochondria and other calcium buffers of squid axon studied in situ. J. Gen. Physiol. 72:101–127. 10.1085/jgp.72.1.101 702105 PMC2228518

[bib23] Broussard, G.J., and L. Petreanu. 2021. Eavesdropping wires: Recording activity in axons using genetically encoded calcium indicators. J. Neurosci. Methods. 360:109251. 10.1016/j.jneumeth.2021.109251 34119572 PMC8363211

[bib24] Bruton, J., P. Tavi, J. Aydin, H. Westerblad, and J. Lännergren. 2003. Mitochondrial and myoplasmic [Ca2+] in single fibres from mouse limb muscles during repeated tetanic contractions. J. Physiol. 551:179–190. 10.1113/jphysiol.2003.043927 12815178 PMC2343157

[bib25] Butera, G., D. Vecellio Reane, M. Canato, L. Pietrangelo, S. Boncompagni, F. Protasi, R. Rizzuto, C. Reggiani, and A. Raffaello. 2021. Parvalbumin affects skeletal muscle trophism through modulation of mitochondrial calcium uptake. Cell Rep. 35:109087. 10.1016/j.celrep.2021.109087 33951435 PMC8113653

[bib26] Calì, T., D. Ottolini, A. Negro, and M. Brini. 2012. α-Synuclein controls mitochondrial calcium homeostasis by enhancing endoplasmic reticulum-mitochondria interactions. J. Biol. Chem. 287:17914–17929. 10.1074/jbc.M111.302794 22453917 PMC3365710

[bib27] Canato, M., P. Capitanio, L. Cancellara, L. Leanza, A. Raffaello, D.V. Reane, L. Marcucci, A. Michelucci, F. Protasi, and C. Reggiani. 2019. Excessive accumulation of Ca^2+^ in mitochondria of Y522S-RYR1 knock-in mice: A link between leak from the sarcoplasmic reticulum and altered redox state. Front. Physiol. 10:1142. 10.3389/fphys.2019.01142 31607937 PMC6755340

[bib28] Cannell, M.B., and D.G. Allen. 1984. Model of calcium movements during activation in the sarcomere of frog skeletal muscle. Biophys. J. 45:913–925. 10.1016/S0006-3495(84)84238-1 6733242 PMC1434964

[bib29] Caputo, C., and P. Bolaños. 2008. Effect of mitochondria poisoning by FCCP on Ca^2+^ signaling in mouse skeletal muscle fibers. Pflugers Arch. 455:733–743. 10.1007/s00424-007-0317-0 17676335

[bib30] Carafoli, E., P. Patriarca, and C.S. Rossi. 1969. A comparative study of the role of mitochondria and the sarcoplasmic reticulum in the uptake and release of Ca++ by the rat diaphragm. J. Cell. Physiol. 74:17–30. 10.1002/jcp.1040740104 5799499

[bib31] Carafoli, E., R. Tiozzo, G. Lugli, F. Crovetti, and C. Kratzing. 1974. The release of calcium from heart mitochondria by sodium. J. Mol. Cell. Cardiol. 6:361–371. 10.1016/0022-2828(74)90077-7 4855051

[bib32] Chalmers, S., and D.G. Nicholls. 2003. The relationship between free and total calcium concentrations in the matrix of liver and brain mitochondria. J. Biol. Chem. 278:19062–19070. 10.1074/jbc.M212661200 12660243

[bib33] Chernorudskiy, A.L., and E. Zito. 2017. Regulation of calcium homeostasis by ER redox: A close-up of the ER/mitochondria connection. J. Mol. Biol. 429:620–632. 10.1016/j.jmb.2017.01.017 28137421

[bib34] Collins, T.J., P. Lipp, M.J. Berridge, and M.D. Bootman. 2001. Mitochondrial Ca^2+^ uptake depends on the spatial and temporal profile of cytosolic Ca^2+^ signals. J. Biol. Chem. 276:26411–26420. 10.1074/jbc.M101101200 11333261

[bib35] Crompton, M., M. Capano, and E. Carafoli. 1976. The sodium-induced efflux of calcium from heart mitochondria. Eur. J. Biochem. 69:453–462. 10.1111/j.1432-1033.1976.tb10930.x 923566

[bib36] Crompton, M., A. Costi, and L. Hayat. 1987. Evidence for the presence of a reversible Ca^2+^-dependent pore activated by oxidative stress in heart mitochondria. Biochem. J. 245:915–918. 10.1042/bj2450915 3117053 PMC1148218

[bib37] Csernoch, L., J.C. Bernengo, P. Szentesi, and V. Jacquemond. 1998. Measurements of intracellular Mg^2+^ concentration in mouse skeletal muscle fibers with the fluorescent indicator mag-indo-1. Biophys. J. 75:957–967. 10.1016/S0006-3495(98)77584-8 9675196 PMC1299769

[bib38] David, G., and E.F. Barrett. 2003. Mitochondrial Ca^2+^ uptake prevents desynchronization of quantal release and minimizes depletion during repetitive stimulation of mouse motor nerve terminals. J. Physiol. 548:425–438. 10.1113/jphysiol.2002.035196 12588898 PMC2342850

[bib39] David, G., J.N. Barrett, and E.F. Barrett. 1998. Evidence that mitochondria buffer physiological Ca^2+^ loads in lizard motor nerve terminals. J. Physiol. 509 (Pt1):59–65. 10.1111/j.1469-7793.1998.059bo.x 9547381 PMC2230953

[bib40] De Stefani, D., A. Raffaello, E. Teardo, I. Szabò, and R. Rizzuto. 2011. A forty-kilodalton protein of the inner membrane is the mitochondrial calcium uniporter. Nature. 476:336–340. 10.1038/nature10230 21685888 PMC4141877

[bib41] Debattisti, V., A. Horn, R. Singh, E.L. Seifert, M.W. Hogarth, D.A. Mazala, K.T. Huang, R. Horvath, J.K. Jaiswal, and G. Hajnóczky. 2019. Dysregulation of mitochondrial Ca^2+^ uptake and sarcolemma repair underlie muscle weakness and wasting in patients and mice lacking MICU1. Cell Rep. 29:1274–1286.e6. 10.1016/j.celrep.2019.09.063 31665639 PMC7007691

[bib42] DeLuca, H.F., and G.W. Engstrom. 1961. Calcium uptake by rat kidney mitochondria. Proc. Natl. Acad. Sci. USA. 47:1744–1750. 10.1073/pnas.47.11.1744 13885269 PMC223205

[bib43] Denton, R.M., J.G. McCormack, and N.J. Edgell. 1980. Role of calcium ions in the regulation of intramitochondrial metabolism. Effects of Na^+^, Mg^2+^ and ruthenium red on the Ca^2+^-stimulated oxidation of oxoglutarate and on pyruvate dehydrogenase activity in intact rat heart mitochondria. Biochem. J. 190:107–117. 10.1042/bj1900107 6160850 PMC1162068

[bib128] Denton, R.M., D.A. Richards, and J.G. Chin. 1978. Calcium ions and the regulation of NAD^+^-linked isocitrate dehydrogenase from the mitochondria of rat heart and other tissues. Biochem. J. 176:899–906. 10.1042/bj1760899 218557 PMC1186314

[bib44] Denton, R.M., P.J. Randle, and B.R. Martin. 1972. Stimulation by calcium ions of pyruvate dehydrogenase phosphate phosphatase. Biochem. J. 128:161–163. 10.1042/bj1280161 4343661 PMC1173580

[bib45] Dirksen, R.T. 2009. Sarcoplasmic reticulum-mitochondrial through-space coupling in skeletal muscle. Appl. Physiol. Nutr. Metab. 34:389–395. 10.1139/H09-044 19448704 PMC2748314

[bib46] Dolphin, A.C. 2021. Functions of presynaptic voltage-gated calcium channels. Function. 2:zqaa027. 10.1093/function/zqaa027 33313507 PMC7709543

[bib47] Dong, Z., S. Shanmughapriya, D. Tomar, N. Siddiqui, S. Lynch, N. Nemani, S.L. Breves, X. Zhang, A. Tripathi, P. Palaniappan, . 2017. Mitochondrial Ca^2+^ uniporter is a mitochondrial luminal redox sensor that augments MCU channel activity. Mol. Cell. 65:1014–1028.e7. 10.1016/j.molcel.2017.01.032 28262504 PMC5357178

[bib48] Drago, I., D. De Stefani, R. Rizzuto, and T. Pozzan. 2012. Mitochondrial Ca^2+^ uptake contributes to buffering cytoplasmic Ca^2+^ peaks in cardiomyocytes. Proc. Natl. Acad. Sci. USA. 109:12986–12991. 10.1073/pnas.1210718109 22822213 PMC3420165

[bib49] Eisner, D.A., J.L. Caldwell, K. Kistamás, and A.W. Trafford. 2017. Calcium and excitation-contraction coupling in the heart. Circ. Res. 121:181–195. 10.1161/CIRCRESAHA.117.310230 28684623 PMC5497788

[bib50] Eisner, V., G. Lenaers, and G. Hajnóczky. 2014. Mitochondrial fusion is frequent in skeletal muscle and supports excitation-contraction coupling. J. Cell. Biol. 205:179–195. 10.1083/jcb.201312066 24751540 PMC4003250

[bib51] Favaro, G., V. Romanello, T. Varanita, M. Andrea Desbats, V. Morbidoni, C. Tezze, M. Albiero, M. Canato, G. Gherardi, D. De Stefani, . 2019. DRP1-mediated mitochondrial shape controls calcium homeostasis and muscle mass. Nat. Commun. 10:2576. 10.1038/s41467-019-10226-9 31189900 PMC6561930

[bib52] Favaron, M., and P. Bernardi. 1985. Tissue-specific modulation of the mitochondrial calcium uniporter by magnesium ions. FEBS Lett. 183:260–264. 10.1016/0014-5793(85)80789-4 3987891

[bib53] Feno, S., G. Butera, D. Vecellio Reane, R. Rizzuto, and A. Raffaello. 20192019. Crosstalk between calcium and ROS in pathophysiological conditions. Oxid. Med. Cell. Longev. 2019:9324018. 10.1155/2019/9324018 PMC6507098 31178978

[bib54] Fernandez-Sanz, C., S. De la Fuente, and S.-S. Sheu. 2019. Mitochondrial Ca^2+^ concentrations in live cells: Quantification methods and discrepancies. FEBS Lett. 593:1528–1541. 10.1002/1873-3468.13427 31058316 PMC7573507

[bib55] Fieni, F., S.B. Lee, Y.N. Jan, and Y. Kirichok. 2012. Activity of the mitochondrial calcium uniporter varies greatly between tissues. Nat. Commun. 3:1317. 10.1038/ncomms2325 23271651 PMC3818247

[bib134] Fontaine, E., O. Eriksson, F. Ichas, and P. Bernardi. 1998. Regulation of the permeability transition pore in skeletal muscle mitochondria. Modulation By electron flow through the respiratory chain complex i. J. Biol. Chem. 273(20):12662–12668. 10.1074/jbc.273.20.12662 9575229

[bib56] Franzini-Armstrong, C. 1984. Freeze-fracture of frog slow tonic fibers. Structure of surface and internal membranes. Tissue Cell. 16:647–664. 10.1016/0040-8166(84)90038-7 6333092

[bib57] Friel, D.D., and R.W. Tsien. 1992. A caffeine- and ryanodine-sensitive Ca^2+^ store in bullfrog sympathetic neurones modulates effects of Ca^2+^ entry on [Ca2+]i. J. Physiol. 450:217–246. 10.1113/jphysiol.1992.sp019125 1432708 PMC1176120

[bib58] Fryer, M.W., and D.G. Stephenson. 1996. Total and sarcoplasmic reticulum calcium contents of skinned fibres from rat skeletal muscle. J. Physiol. 493 (Pt 2):357–370. 10.1113/jphysiol.1996.sp021388 8782101 PMC1158922

[bib59] García-Chacón, L.E., K.T. Nguyen, G. David, and E.F. Barrett. 2006. Extrusion of Ca^2+^ from mouse motor terminal mitochondria via a Na^+^–Ca^2+^ exchanger increases post-tetanic evoked release. J. Physiol. 574:663–675. 10.1113/jphysiol.2006.110841 16613870 PMC1817729

[bib60] Gherardi, G., L. Nogara, S. Ciciliot, G.P. Fadini, B. Blaauw, P. Braghetta, P. Bonaldo, D. De Stefani, R. Rizzuto, and C. Mammucari. 2019. Loss of mitochondrial calcium uniporter rewires skeletal muscle metabolism and substrate preference. Cell Death Differ. 26:362–381. 10.1038/s41418-018-0191-7 30232375 PMC6329801

[bib61] Giacomello, M., I. Drago, M. Bortolozzi, M. Scorzeto, A. Gianelle, P. Pizzo, and T. Pozzan. 2010. Ca^2+^ hot spots on the mitochondrial surface are generated by Ca^2+^ mobilization from stores, but not by activation of store-operated Ca^2+^ channels. Mol. Cell. 38:280–290. 10.1016/j.molcel.2010.04.003 20417605

[bib62] Gillis, J.M. 1997. Inhibition of mitochondrial calcium uptake slows down relaxation in mitochondria-rich skeletal muscles. J. Muscle Res. Cell Motil. 18:473–483. 10.1023/a:1018603032590 9276340

[bib63] Griffiths, E.J., and A.P. Halestrap. 1995. Mitochondrial non-specific pores remain closed during cardiac ischaemia, but open upon reperfusion. Biochem. J. 307 (Pt 1):93–98. 10.1042/bj3070093 7717999 PMC1136749

[bib64] Holash, R.J., and B.R. MacIntosh. 2019. A stochastic simulation of skeletal muscle calcium transients in a structurally realistic sarcomere model using MCell. PLoS Comput. Biol. 15:e1006712. 10.1371/journal.pcbi.1006712 30845143 PMC6424466

[bib65] Holmström, K.M., X. Pan, J.C. Liu, S. Menazza, J. Liu, T.T. Nguyen, H. Pan, R.J. Parks, S. Anderson, A. Noguchi, . 2015. Assessment of cardiac function in mice lacking the mitochondrial calcium uniporter. J. Mol. Cell Cardiol. 85:178–182. 10.1016/j.yjmcc.2015.05.022 26057074 PMC4530042

[bib66] Horn, A., J.H. VanderMeulen, A. Defour, M. Hogarth, S.C. Sreetama, A. Reed, L. Scheffer, N.S. Chandel, and J.K. Jaiswal. 2017. Mitochondrial redox signaling enables repair of injured skeletal muscle cells. Sci. Signal. 10:eaaj1978. 10.1126/scisignal.aaj1978 28874604 PMC5949579

[bib67] Hunter, D.R., R.A. Haworth, and J.H. Southard. 1976. Relationship between configuration, function, and permeability in calcium-treated mitochondria. J. Biol. Chem. 251:5069–5077. 10.1016/s0021-9258(17)33220-9 134035

[bib68] Hutson, S.M., D.R. Pfeiffer, and H.A. Lardy. 1976. Effect of cations and anions on the steady state kinetics of energy-dependent Ca^2+^ transport in rat liver mitochondria. J. Biol. Chem. 251:5251–5258. 10.1016/S0021-9258(17)33154-X 783158

[bib69] Irwin, W.A., N. Bergamin, P. Sabatelli, C. Reggiani, A. Megighian, L. Merlini, P. Braghetta, M. Columbaro, D. Volpin, G.M. Bressan, . 2003. Mitochondrial dysfunction and apoptosis in myopathic mice with collagen VI deficiency. Nat. Genet. 35:367–371. 10.1038/ng1270 14625552

[bib70] Ivanova, H., T. Vervliet, G. Monaco, L.E. Terry, N. Rosa, M.R. Baker, J.B. Parys, I.I. Serysheva, D.I. Yule, and G. Bultynck. 2020. Bcl-2-Protein family as modulators of IP3 receptors and other organellar Ca^2+^ channels. Cold Spring Harb. Perspect. Biol. 12:a035089. 10.1101/cshperspect.a035089 31501195 PMC7111250

[bib71] Jaconi, M., C. Bony, S.M. Richards, A. Terzic, S. Arnaudeau, G. Vassort, and M. Pucéat. 2000. Inositol 1, 4, 5-trisphosphate directs Ca^2+^ flow between mitochondria and the endoplasmic/sarcoplasmic reticulum: A role in regulating cardiac autonomic Ca^2+^ spiking. Mol. Biol. Cell. 11:1845–1858. 10.1091/mbc.11.5.1845 10793156 PMC14888

[bib72] Jou, M., and S. Sheu. 1994. Mitochondrial Ca^2+^ oscillations in single living cells revealed by rhod-2 and laser confocal microscopy. Biophys. J. 66:A94–A94

[bib73] Jou, M.J., T.I. Peng, and S.S. Sheu. 1996. Histamine induces oscillations of mitochondrial free Ca^2+^ concentration in single cultured rat brain astrocytes. J. Physiol. 497 (Pt 2):299–308. 10.1113/jphysiol.1996.sp021769 8961176 PMC1160985

[bib74] Kwong, J.Q., X. Lu, R.N. Correll, J.A. Schwanekamp, R.J. Vagnozzi, M.A. Sargent, A.J. York, J. Zhang, D.M. Bers, and J.D. Molkentin. 2015. The mitochondrial calcium uniporter selectively matches metabolic output to acute contractile stress in the heart. Cell Rep. 12:15–22. 10.1016/j.celrep.2015.06.002 26119742 PMC4497842

[bib75] Lamboley, C.R., L. Pearce, C. Seng, A. Meizoso-Huesca, D.P. Singh, B.P. Frankish, V. Kaura, H.P. Lo, C. Ferguson, P.D. Allen, . 2021. Ryanodine receptor leak triggers fiber Ca^2+^ redistribution to preserve force and elevate basal metabolism in skeletal muscle. Sci. Adv. 7:eabi7166. 10.1126/sciadv.abi7166 34705503 PMC8550231

[bib76] Lamboley, C.R., S.A. Kake Guena, F. Touré, C. Hébert, L. Yaddaden, S. Nadeau, P. Bouchard, L. Wei-LaPierre, J. Lainé, E.C. Rousseau, . 2015. New method for determining total calcium content in tissue applied to skeletal muscle with and without calsequestrin. J. Gen. Physiol. 145:127–153. 10.1085/jgp.201411250 25624449 PMC4306712

[bib77] Lännergren, J., H. Westerblad, and J.D. Bruton. 2001. Changes in mitochondrial Ca^2+^ detected with Rhod-2 in single frog and mouse skeletal muscle fibres during and after repeated tetanic contractions. J. Muscle Res. Cell Motil. 22:265–275. 10.1023/a:1012227009544 11763199

[bib78] Lu, X., K.S. Ginsburg, S. Kettlewell, J. Bossuyt, G.L. Smith, and D.M. Bers. 2013. Measuring local gradients of intramitochondrial [Ca(2+)] in cardiac myocytes during sarcoplasmic reticulum Ca(2+) release. Circ. Res. 112:424–431. 10.1161/CIRCRESAHA.111.300501 23243207 PMC3566246

[bib79] Luongo, T.S., J.P. Lambert, P. Gross, M. Nwokedi, A.A. Lombardi, S. Shanmughapriya, A.C. Carpenter, D. Kolmetzky, E. Gao, J.H. van Berlo, . 2017. The mitochondrial Na^+^/Ca^2+^ exchanger is essential for Ca^2+^ homeostasis and viability. Nature. 545:93–97. 10.1038/nature22082 28445457 PMC5731245

[bib80] Luongo, T.S., J.P. Lambert, A. Yuan, X. Zhang, P. Gross, J. Song, S. Shanmughapriya, E. Gao, M. Jain, S.R. Houser, . 2015. The mitochondrial calcium uniporter matches energetic supply with cardiac workload during stress and modulates permeability transition. Cell Rep. 12:23–34. 10.1016/j.celrep.2015.06.017 26119731 PMC4517182

[bib81] Madsen, K., P. Ertbjerg, M.S. Djurhuus, and P.K. Pedersen. 1996. Calcium content and respiratory control index of skeletal muscle mitochondria during exercise and recovery. Am. J. Physiol. 271:E1044–E1050. 10.1152/ajpendo.1996.271.6.E1044 8997224

[bib82] Magnus, G., and J. Keizer. 1997. Minimal model of beta-cell mitochondrial Ca^2+^ handling. Am. J. Physiol. 273:C717–C733. 10.1152/ajpcell.1997.273.2.C717 9277370

[bib83] Mallilankaraman, K., C. Cárdenas, P.J. Doonan, H.C. Chandramoorthy, K.M. Irrinki, T. Golenár, G. Csordás, P. Madireddi, J. Yang, M. Müller, . 2012. MCUR1 is an essential component of mitochondrial Ca^2+^ uptake that regulates cellular metabolism. Nat. Cell Biol. 14:1336–1343. 10.1038/ncb2622 23178883 PMC3511605

[bib84] Mammucari, C., G. Gherardi, I. Zamparo, A. Raffaello, S. Boncompagni, F. Chemello, S. Cagnin, A. Braga, S. Zanin, G. Pallafacchina, . 2015. The mitochondrial calcium uniporter controls skeletal muscle trophism in vivo. Cell Rep. 10:1269–1279. 10.1016/j.celrep.2015.01.056 25732818 PMC4351162

[bib85] Manno, C., M. Sztretye, L. Figueroa, P.D. Allen, and E. Ríos. 2013. Dynamic measurement of the calcium buffering properties of the sarcoplasmic reticulum in mouse skeletal muscle. J. Physiol. 591:423–442. 10.1113/jphysiol.2012.243444 23148320 PMC3577525

[bib86] Marban, E., T.J. Rink, R.W. Tsien, and R.Y. Tsien. 1980. Free calcium in heart muscle at rest and during contraction measured with Ca^2+^ -sensitive microelectrodes. Nature. 286:845–850. 10.1038/286845a0 7412868

[bib87] Marcucci, L., M. Canato, F. Protasi, G.J.M. Stienen, and C. Reggiani. 2018. A 3D diffusional-compartmental model of the calcium dynamics in cytosol, sarcoplasmic reticulum and mitochondria of murine skeletal muscle fibers. PLoS One. 13:e0201050. 10.1371/journal.pone.0201050 30048500 PMC6062086

[bib132] McMillin-Wood, J., P.E. Wolkowicz, A. Chu, C.A. Tate, M.A. Goldstein, and M.L. Entman. 1980. Calcium uptake by two preparations of mitochondria from heart. Biochim. Biophys. Acta. 591:251–265. 10.1016/0005-2728(80)90157-7 7397124

[bib88] Miyata, H., H.S. Silverman, S.J. Sollott, E.G. Lakatta, M.D. Stern, and R.G. Hansford. 1991. Measurement of mitochondrial free Ca^2+^ concentration in living single rat cardiac myocytes. Am. J. Physiol. 261:H1123–H1134. 10.1152/ajpheart.1991.261.4.H1123 1928394

[bib89] Miyawaki, A., J. Llopis, R. Heim, J.M. McCaffery, J.A. Adams, M. Ikura, and R.Y. Tsien. 1997. Fluorescent indicators for Ca^2+^ based on green fluorescent proteins and calmodulin. Nature. 388:882–887. 10.1038/42264 9278050

[bib90] Montero, M., M.T. Alonso, E. Carnicero, I. Cuchillo-Ibáñez, A. Albillos, A.G. García, J. García-Sancho, and J. Alvarez. 2000. Chromaffin-cell stimulation triggers fast millimolar mitochondrial Ca^2+^ transients that modulate secretion. Nat. Cell Biol. 2:57–61. 10.1038/35000001 10655583

[bib91] Nicholls, D.G. 1978. The regulation of extramitochondrial free calcium ion concentration by rat liver mitochondria. Biochem. J. 176:463–474. 10.1042/bj1760463 33670 PMC1186255

[bib92] Padmaraj, D., R. Pande, J.H. Miller, J. Wosik, and W. Zagozdzon-Wosik. 2014. Mitochondrial membrane studies using impedance spectroscopy with parallel pH monitoring. PLoS One. 9:e101793. 10.1371/journal.pone.0101793 25010497 PMC4091947

[bib93] Palty, R., W.F. Silverman, M. Hershfinkel, T. Caporale, S.L. Sensi, J. Parnis, C. Nolte, D. Fishman, V. Shoshan-Barmatz, S. Herrmann, . 2010. NCLX is an essential component of mitochondrial Na^+^/Ca^2+^ exchange. Proc. Natl. Acad. Sci. USA. 107:436–441. 10.1073/pnas.0908099107 20018762 PMC2806722

[bib94] Patron, M., V. Granatiero, J. Espino, R. Rizzuto, and D. De Stefani. 2019. MICU3 is a tissue-specific enhancer of mitochondrial calcium uptake. Cell Death Differ. 26:179–195. 10.1038/s41418-018-0113-8 29725115 PMC6124646

[bib95] Perocchi, F., V.M. Gohil, H.S. Girgis, X.R. Bao, J.E. McCombs, A.E. Palmer, and V.K. Mootha. 2010. MICU1 encodes a mitochondrial EF hand protein required for Ca^2+^ uptake. Nature. 467:291–296. 10.1038/nature09358 20693986 PMC2977980

[bib96] Pinton, P., and R. Rizzuto. 2006. Bcl-2 and Ca^2+^ homeostasis in the endoplasmic reticulum. Cell Death Differ. 13:1409–1418. 10.1038/sj.cdd.4401960 16729032

[bib97] Plovanich, M., R.L. Bogorad, Y. Sancak, K.J. Kamer, L. Strittmatter, A.A. Li, H.S. Girgis, S. Kuchimanchi, J. De Groot, L. Speciner, . 2013. MICU2, a paralog of MICU1, resides within the mitochondrial uniporter complex to regulate calcium handling. PLoS One. 8:e55785. 10.1371/journal.pone.0055785 23409044 PMC3567112

[bib98] Raffaello, A., D. De Stefani, D. Sabbadin, E. Teardo, G. Merli, A. Picard, V. Checchetto, S. Moro, I. Szabò, and R. Rizzuto. 2013. The mitochondrial calcium uniporter is a multimer that can include a dominant-negative pore-forming subunit. EMBO J. 32:2362–2376. 10.1038/emboj.2013.157 23900286 PMC3771344

[bib131] Reed, K.C., and F.L. Bygrave. 1975. A kinetic study of mitochondrial calcium transport. Eur. J. Biochem. 55(3):497–504. 10.1111/j.1432-1033.1975.tb02187.x 240699

[bib99] Rincón, O.A., A.F. Milán, J.C. Calderón, and M.A. Giraldo. 2021. Comprehensive simulation of Ca^2+^ transients in the continuum of mouse skeletal muscle fiber types. Int. J. Mol. Sci. 22:12378. 10.3390/ijms222212378 34830262 PMC8624975

[bib100] Ríos, E. 2010. The cell boundary theorem: A simple law of the control of cytosolic calcium concentration. J. Physiol. Sci. 60:81–84. 10.1007/s12576-009-0069-z 19937486 PMC2821834

[bib101] Rizzuto, R., C. Bastianutto, M. Brini, M. Murgia, and T. Pozzan. 1994. Mitochondrial Ca^2+^ homeostasis in intact cells. J. Cell. Biol. 126:1183–1194. 10.1083/jcb.126.5.1183 8063855 PMC2120160

[bib102] Rizzuto, R., M. Brini, M. Murgia, and T. Pozzan. 1993. Microdomains with high Ca^2+^ close to IP3-sensitive channels that are sensed by neighboring mitochondria. Science. 262:744–747. 10.1126/science.8235595 8235595

[bib103] Rizzuto, R., A.W. Simpson, M. Brini, and T. Pozzan. 1992. Rapid changes of mitochondrial Ca^2+^ revealed by specifically targeted recombinant aequorin. Nature. 358:325–327. 10.1038/358325a0 1322496

[bib104] Robert, V., P. Gurlini, V. Tosello, T. Nagai, A. Miyawaki, F. Di Lisa, and T. Pozzan. 2001. Beat-to-beat oscillations of mitochondrial [Ca2+] in cardiac cells. EMBO J. 20:4998–5007. 10.1093/emboj/20.17.4998 11532963 PMC125611

[bib105] Rudolf, R., M. Mongillo, P.J. Magalhães, and T. Pozzan. 2004. In vivo monitoring of Ca^2+^ uptake into mitochondria of mouse skeletal muscle during contraction. J. Cell. Biol. 166:527–536. 10.1083/jcb.200403102 15314066 PMC2172216

[bib106] Sancak, Y., A.L. Markhard, T. Kitami, E. Kovács-Bogdán, K.J. Kamer, N.D. Udeshi, S.A. Carr, D. Chaudhuri, D.E. Clapham, A.A. Li, . 2013. EMRE is an essential component of the mitochondrial calcium uniporter complex. Science. 342:1379–1382. 10.1126/science.1242993 24231807 PMC4091629

[bib107] Sanchez, C., C. Berthier, Y. Tourneur, L. Monteiro, B. Allard, L. Csernoch, and V. Jacquemond. 2021. Detection of Ca^2+^ transients near ryanodine receptors by targeting fluorescent Ca^2+^ sensors to the triad. J. Gen. Physiol. 153:e202012592. 10.1085/jgp.202012592 33538764 PMC7868779

[bib108] Sander, P., T. Gudermann, and J. Schredelseker. 2021. A calcium guard in the outer membrane: Is VDAC a regulated gatekeeper of mitochondrial calcium uptake? Int. J. Mol. Sci. 22:946. 10.3390/ijms22020946 33477936 PMC7833399

[bib130] Scarpa, A., and P. Graziotti. 1973. Mechanisms for intracellular calcium regulation in heart. I. Stopped-flow measurements of Ca++ uptake by cardiac mitochondria. J. Gen. Physiol. 62(6):756–772. 10.1085/jgp.62.6.756 4548716 PMC2226144

[bib109] Schneggenburger, R., and E. Neher. 2005. Presynaptic calcium and control of vesicle fusion. Curr. Opin. Neurobiol. 15:266–274. 10.1016/j.conb.2005.05.006 15919191

[bib110] Schwerzmann, K., H. Hoppeler, S.R. Kayar, and E.R. Weibel. 1989. Oxidative capacity of muscle and mitochondria: Correlation of physiological, biochemical, and morphometric characteristics. Proc. Natl. Acad. Sci. USA. 86:1583–1587. 10.1073/pnas.86.5.1583 2922400 PMC286742

[bib111] Scorzeto, M., M. Giacomello, L. Toniolo, M. Canato, B. Blaauw, C. Paolini, F. Protasi, C. Reggiani, and G.J.M. Stienen. 2013. Mitochondrial Ca^2+^-handling in fast skeletal muscle fibers from wild type and calsequestrin-null mice. PLoS One. 8:e74919. 10.1371/journal.pone.0074919 24098358 PMC3789688

[bib112] Sembrowich, W.L., J.J. Quintinskie, and G. Li. 1985. Calcium uptake in mitochondria from different skeletal muscle types. J. Appl Physiol. 59:137–141. 10.1152/jappl.1985.59.1.137 4030557

[bib113] Somlyo, A.P., and A.V. Somlyo. 1986. Electron probe analysis of calcium content and movements in sarcoplasmic reticulum, endoplasmic reticulum, mitochondria, and cytoplasm. J. Cardiovasc. Pharmacol. 8:S42–S47. 10.1097/00005344-198600088-00009 2433524

[bib114] Sun, Q.-A., B. Wang, M. Miyagi, D.T. Hess, and J.S. Stamler. 2013. Oxygen-coupled redox regulation of the skeletal muscle ryanodine receptor/Ca^2+^ release channel (RyR1): Sites and nature of oxidative modification. J. Biol. Chem. 288:22961–22971. 10.1074/jbc.M113.480228 23798702 PMC3743473

[bib115] Tinel, H., J.M. Cancela, H. Mogami, J.V. Gerasimenko, O.V. Gerasimenko, A.V. Tepikin, and O.H. Petersen. 1999. Active mitochondria surrounding the pancreatic acinar granule region prevent spreading of inositol trisphosphate-evoked local cytosolic Ca^2+^ signals. EMBO J. 18:4999–5008. 10.1093/emboj/18.18.4999 10487752 PMC1171571

[bib116] Tsien, R.Y., and T.J. Rink. 1980. Neutral carrier ion-selective microelectrodes for measurement of intracellular free calcium. Biochim. Biophys. Acta. 599:623–638. 10.1016/0005-2736(80)90205-9 7407109

[bib117] Turrens, J.F. 2003. Mitochondrial formation of reactive oxygen species. J. Physiol. 552:335–344. 10.1113/jphysiol.2003.049478 14561818 PMC2343396

[bib118] Vajda, S., M. Mándi, C. Konràd, G. Kiss, A. Ambrus, V. Adam-Vizi, and C. Chinopoulos. 2009. A re-evaluation of the role of matrix acidification in uncoupler-induced Ca^2+^ release from mitochondria. FEBS J. 276:2713–2724. 10.1111/j.1742-4658.2009.06995.x 19459934

[bib119] Vasington, F.D., and J.V. Murphy. 1962. Ca ion uptake by rat kidney mitochondria and its dependence on respiration and phosphorylation. J. Biol. Chem. 237:2670–2677. 10.1016/s0021-9258(19)73805-8 13925019

[bib120] Vecellio Reane, D., F. Vallese, V. Checchetto, L. Acquasaliente, G. Butera, V. De Filippis, I. Szabò, G. Zanotti, R. Rizzuto, and A. Raffaello. 2016. A MICU1 splice variant confers high sensitivity to the mitochondrial Ca^2+^ uptake machinery of skeletal muscle. Mol. Cell. 64:760–773. 10.1016/j.molcel.2016.10.001 27818145

[bib127] Vinogradov, A., and A. Scarpa. 1973. The initial velocities of calcium uptake by rat liver mitochondria. J. Biol. Chem. 248(15):5527–5531. 4768910

[bib121] Wang, L., X. Yang, S. Li, Z. Wang, Y. Liu, J. Feng, Y. Zhu, and Y. Shen. 2014. Structural and mechanistic insights into MICU1 regulation of mitochondrial calcium uptake. EMBO J. 33:594–604. 10.1002/embj.201386523 24514027 PMC3989653

[bib122] Wei, A.-C., T. Liu, R.L. Winslow, and B. O’Rourke. 2012. Dynamics of matrix-free Ca^2+^ in cardiac mitochondria: Two components of Ca^2+^ uptake and role of phosphate buffering. J. Gen. Physiol. 139:465–478. 10.1085/jgp.201210784 22641641 PMC3362519

[bib123] Weiss, N., T. Andrianjafiniony, S. Dupré-Aucouturier, S. Pouvreau, D. Desplanches, and V. Jacquemond. 2010. Altered myoplasmic Ca^2+^ handling in rat fast-twitch skeletal muscle fibres during disuse atrophy. Pflugers Arch. 459:631–644. 10.1007/s00424-009-0764-x 19997852

[bib124] Wüst, R.C.I., M. Helmes, J.L. Martin, T.J.T. van der Wardt, R.J.P. Musters, J. van der Velden, and G.J.M. Stienen. 2017. Rapid frequency-dependent changes in free mitochondrial calcium concentration in rat cardiac myocytes: Mitochondrial calcium handling. J. Physiol. 595:2001–2019. 10.1113/JP273589 28028811 PMC5350475

[bib125] Yi, J., C. Ma, Y. Li, N. Weisleder, E. Ríos, J. Ma, and J. Zhou. 2011. Mitochondrial calcium uptake regulates rapid calcium transients in skeletal muscle during excitation-contraction (E-C) coupling. J. Biol. Chem. 286:32436–32443. 10.1074/jbc.M110.217711 21795684 PMC3173159

