# Correction: A controversial issue: Can mitochondria modulate cytosolic calcium and contraction of skeletal muscle fibers?

**DOI:** 10.1085/jgp.20221316709092022c

**Published:** 2022-09-19

**Authors:** Carlo Reggiani, Lorenzo Marcucci

Vol. 154, No. 9 | 10.1085/jgp.202213167 | July 18, 2022

The original version of this paper inadvertantly misrepresented some of the data from the Bruton et al. (2003) reference.

The third paragraph of the Mitochondria in skeletal muscle fibers section has been corrected, and it appears below with the corrections underlined. The errors appear only in PDF versions downloaded on or before September 12, 2022.

In the first decade of the new century, the free Ca^2+^ indicators were applied also in living adult muscle fibers and opened a new perspective. Lännergren et al. (2001) using Rhod2 reported calcium accumulation in mitochondria during prolonged contractile activity in *Xenopus* fibers but not in mouse fast flexor digitorum brevis (FDB) fibers. 2 yr later, Bruton et al. (2003) measuring cytosolic calcium with Indo-1 and mitochondrial calcium with Rhod2 in mouse soleus and EDL fibers observed, after fatiguing stimulation, an increase of mitochondrial matrix calcium in both muscles. The absence of mitochondrial calcium uptake during tetanic stimulation was further confirmed on FDB fibers loaded with Rhod2 (Aydin et al., 2009). In contrast, cameleon probes targeted to mitochondria produced clear-cut evidence of transient increases of mitochondrial Ca^2+^ concentration in electrically stimulated murine muscles with consistent results in tibialis anterior fibers in situ (Rudolf et al., 2004) and in isolated FDB fibers (Scorzeto et al., 2013). The matrix-free Ca^2+^ concentration follows closely cytosolic-free Ca^2+^ variations, even during a single twitch, although with a somewhat different kinetics. A fast rise phase was followed by a slow decrease, and this allowed the integration of the signals coming from repeated stimulations/action potentials. Importantly, the time scale of the metabolic activation triggered by calcium is much faster compared to the activation induced by ADP and creatine which implies energy depletion. Thus, calcium-activation could be interpreted as a feed-forward regulation, while ADP activation appeared as a feed-back control.
